# Supplementary material for: Selective inhibition of cullin 3 neddylation through covalent targeting DCN1 protects mice from acetaminophen-induced liver toxicity
Source: Nat Commun. 2021 May 11;12:2621. doi: 10.1038/s41467-021-22924-4 (PMC8113459; doi:10.1038/s41467-021-22924-4)
Supplement: Supplementary file 1 — Supplementary Information [file 41467_2021_22924_MOESM1_ESM.pdf]

## SUPPLEMENTARY INFORMATION

# Selective inhibition of cullin 3 neddylation through covalent targeting DCN1 protects mice from Acetaminophen-induced liver toxicity

Haibin Zhou[1,8], Jianfeng Lu[1,8], Krishnapriya Chinnaswamy[4], Jeanne Stuckey[4], Liu Liu[1], Donna McEachern[1], Chao-Yie Yang[1], Denzil Bernard[1], Hong Shen[5], Liangyou Rui[5], Yi Sun[6,7], Shaomeng Wang[1,2,3✉]

Departments of [1]Internal Medicine, [2]Pharmacology, [3]Medicinal Chemistry, [4]Life Sciences Institute, [5]Department of Molecular and Integrative Physiology, and [6]Division of Radiation and Cancer Biology, Department of Radiation Oncology, University of Michigan, Ann Arbor, MI 48109, USA

[7]Institute of Translational Medicine, Zhejiang University, Hangzhou, Zhejiang, China

[8]These authors contributed equally

[✉]To whom correspondence should be addressed

Phone: 734-615-0362

Fax: 734-647-9647

E-mail: shaomeng@umich.edu

**SUPPLEMENTARY FIGURES**

The mass spectrum of DCN1 apo-protein:

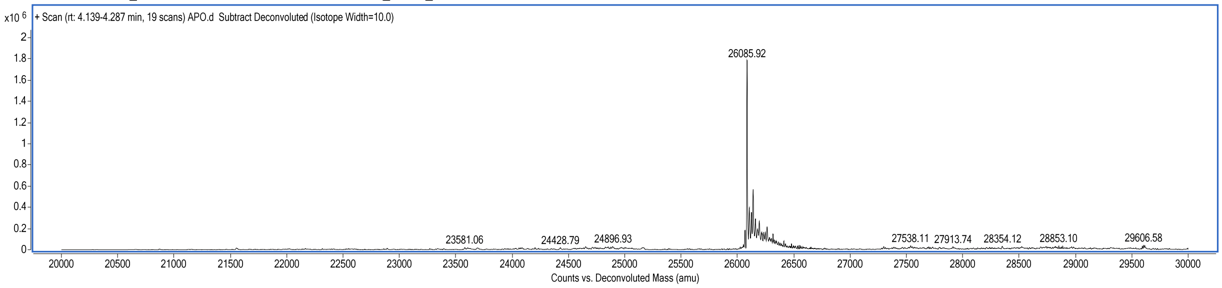


**Supplementary Figure 1. The mass-spectrometric analysis of DCN1 apo-protein.**

The mass spectrum of DCN1 apo-protein incubated with DI-591 (**1**) for 10 min:

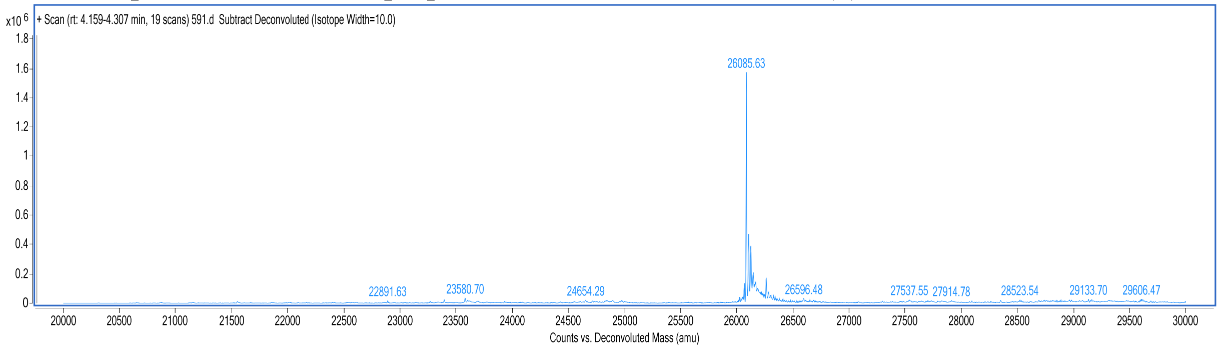


The mass spectrum of DCN1 apo-protein incubated with DI-591 (**1**) for 1 h:

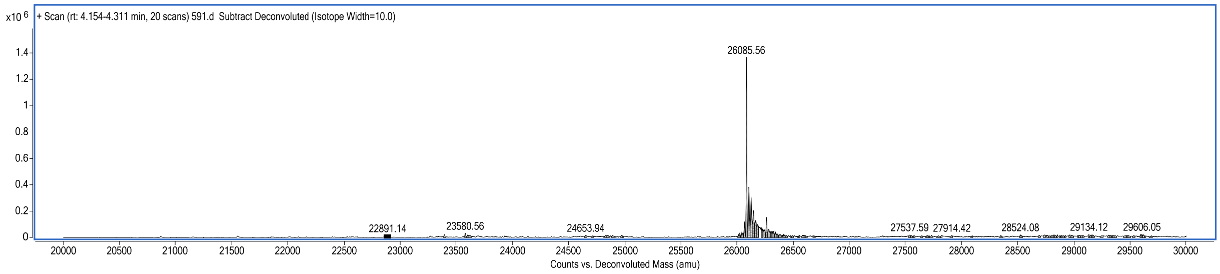


The mass spectrum of DCN1 apo-protein incubated with DI-591 (**1**) for 12 h:

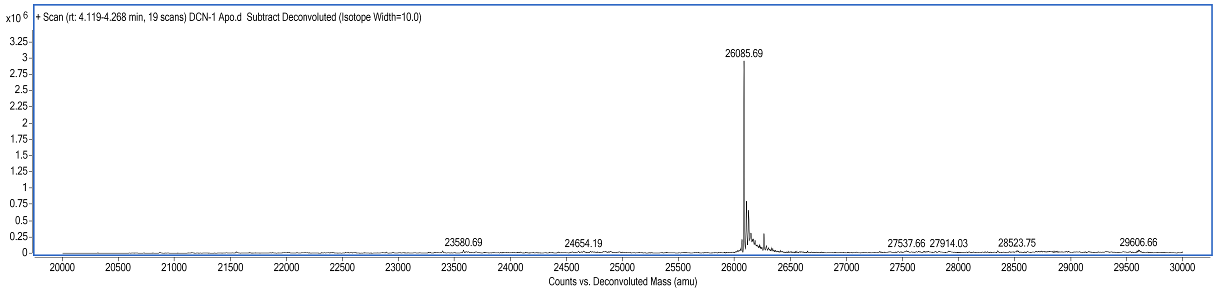


**Supplementary Figure 2. The mass-spectrometric analyses of DCN1 apo-protein incubated with DI-591 (1) for indicated time.**

The mass spectrum of DCN1 apo-protein incubated with compound **2** for 10 min:

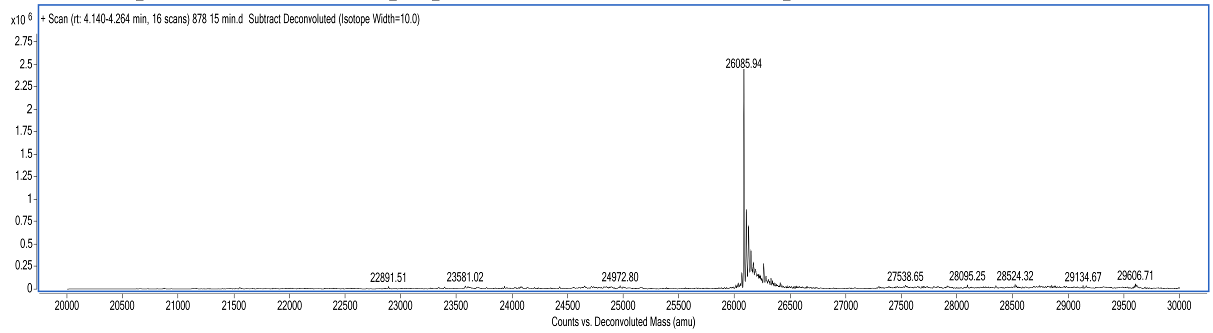


The mass spectrum of DCN1 apo-protein incubated with compound **2** for 1 h:

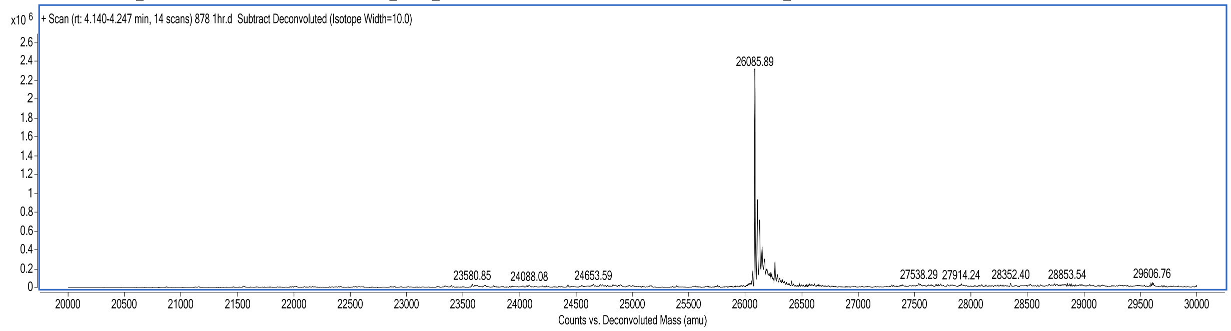


The mass spectrum of DCN1 apo-protein incubated with compound **2** for 3 h:

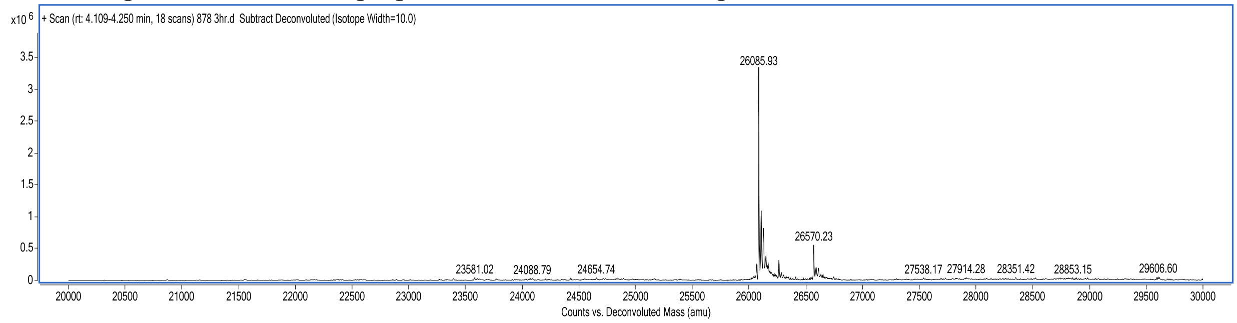


The mass spectrum of DCN1 apo-protein incubated with compound **2** for 12 h:

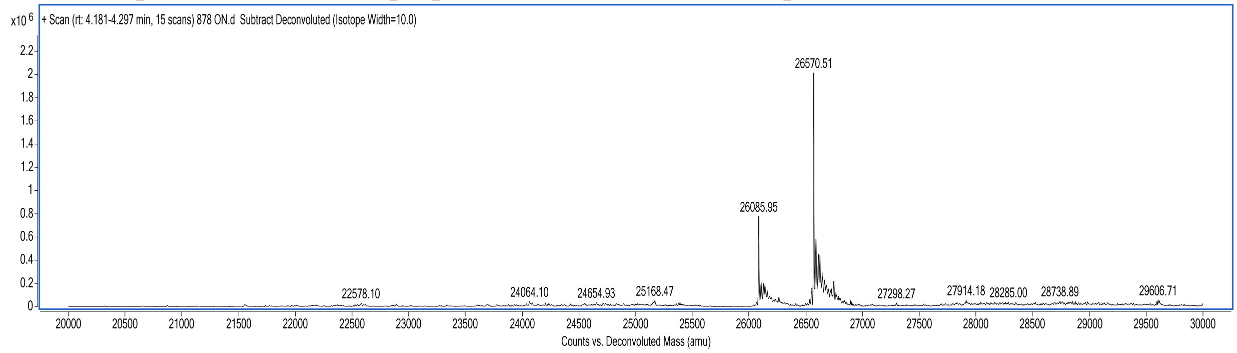


**Supplementary Figure 3. The mass-spectrometric analyses of DCN1 apo-protein incubated with compound 2 for indicated time.**

The mass spectrum of DCN1 apo-protein incubated with compound **3** for 10 min:

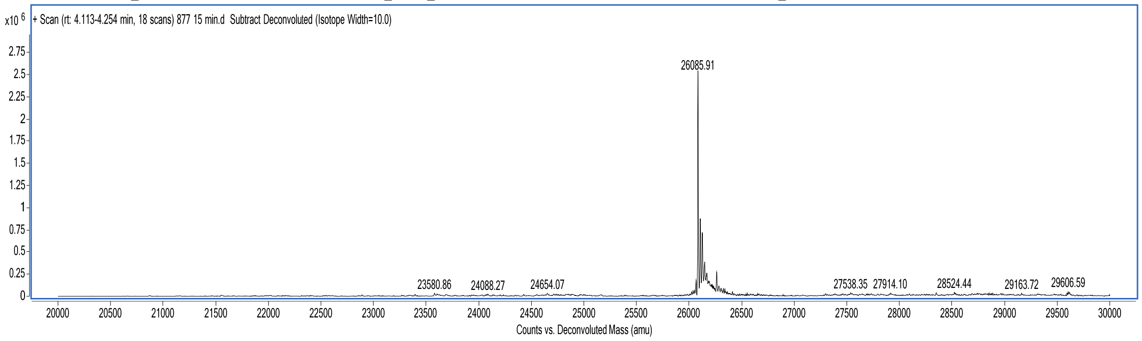


The mass spectrum of DCN1 apo-protein incubated with compound **3** for 1 h:

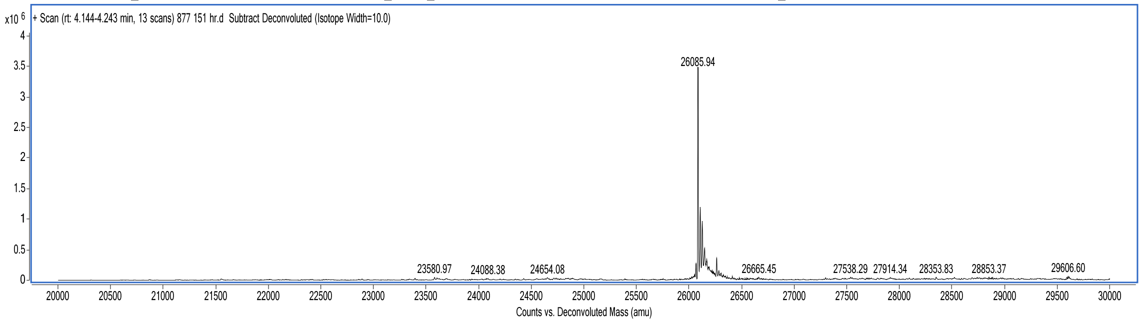


The mass spectrum of DCN1 apo-protein incubated with compound **3** for 3 h:

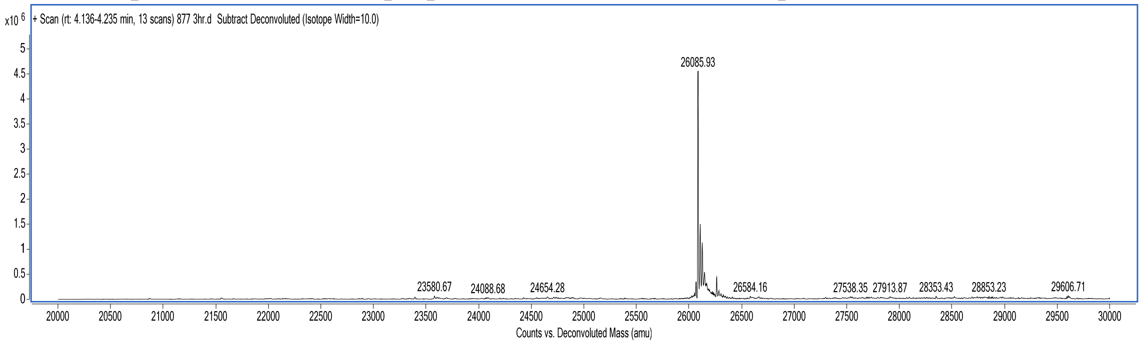


The mass spectrum of DCN1 apo-protein incubated with compound **3** for 12 h:

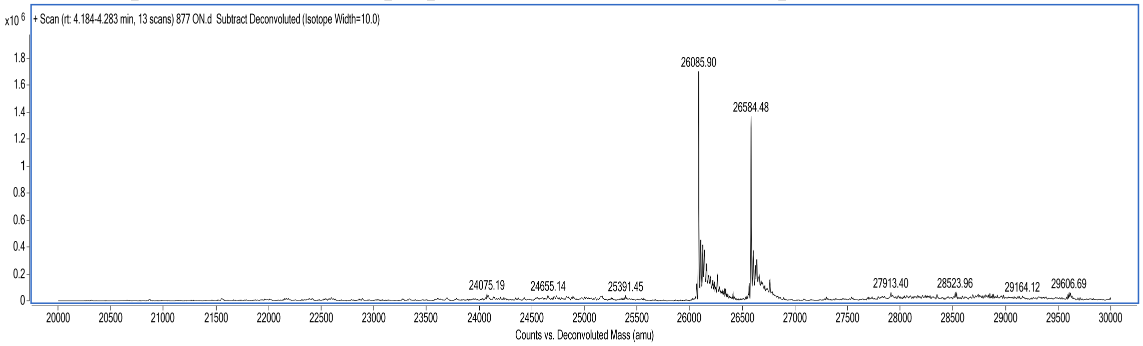


**Supplementary Figure 4. The mass-spectrometric analyses of DCN1 apo-protein incubated with compound 3 for indicated time.**

The mass spectrum of DCN1 apo-protein incubated with compound **4** for 10 min:

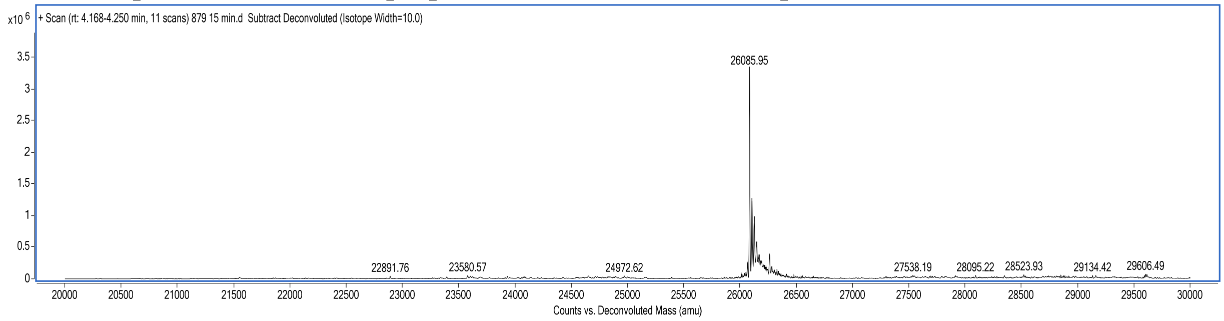


The mass spectrum of DCN1 apo-protein incubated with compound **4** for 1 h:

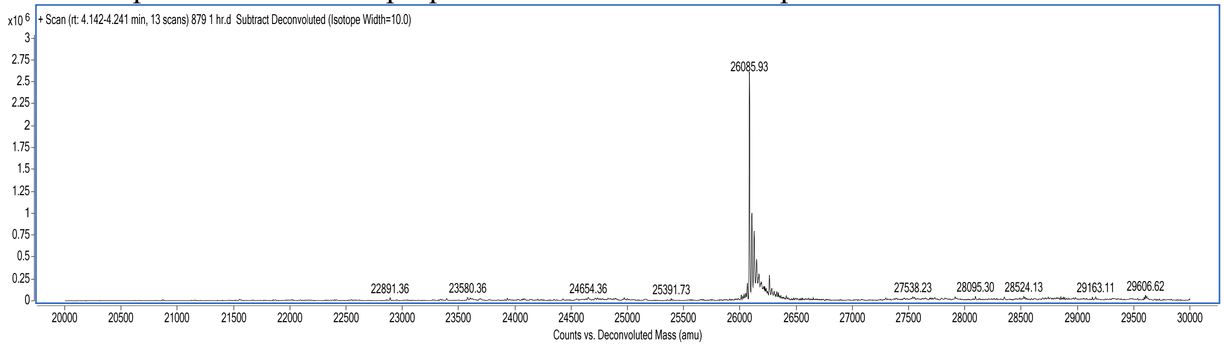


The mass spectrum of DCN1 apo-protein incubated with compound **4** for 3 h:

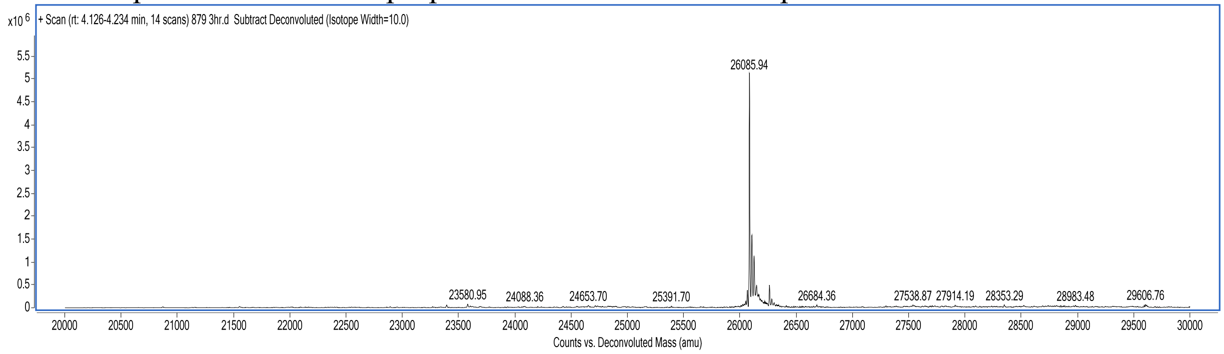


The mass spectrum of DCN1 apo-protein incubated with compound **4** for 12 h:

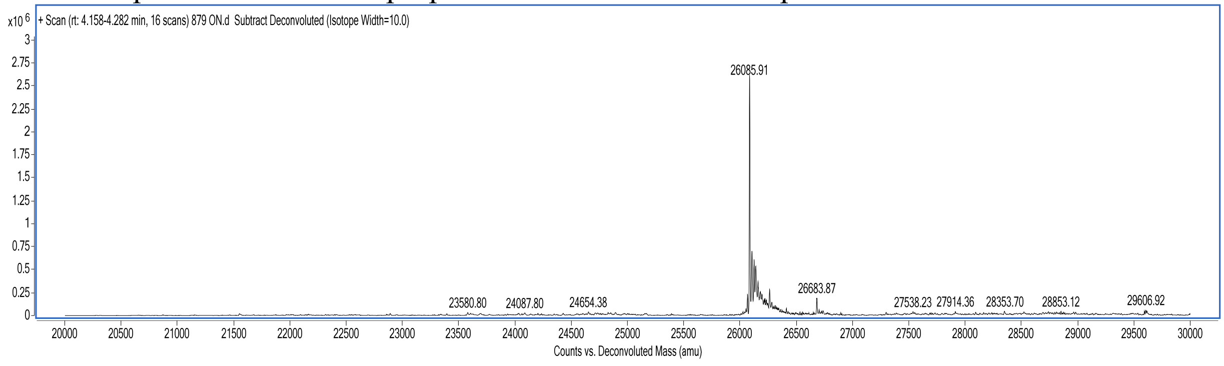


**Supplementary Figure 5. The mass-spectrometric analyses of DCN1 apo-protein incubated with compound 4 for indicated time.**

The mass spectrum of DCN1 apo-protein incubated with compound **5** for 10 min:

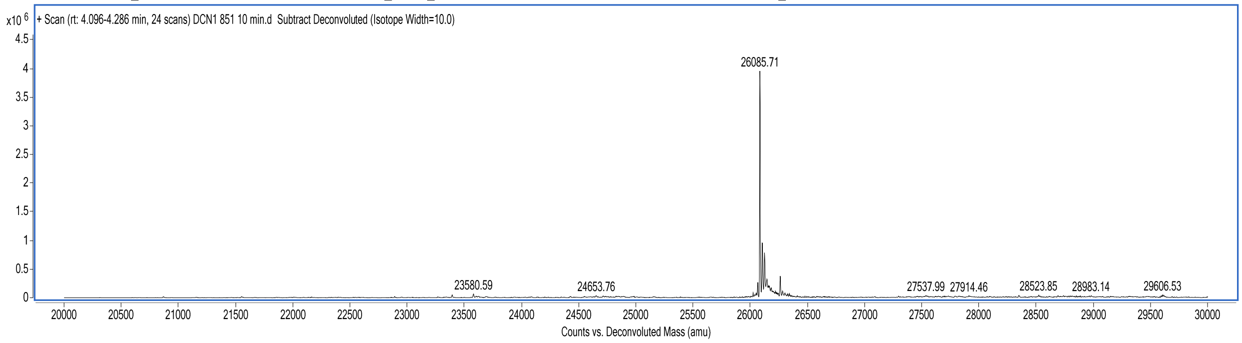


The mass spectrum of DCN1 apo-protein incubated with compound **5** for 1 h:

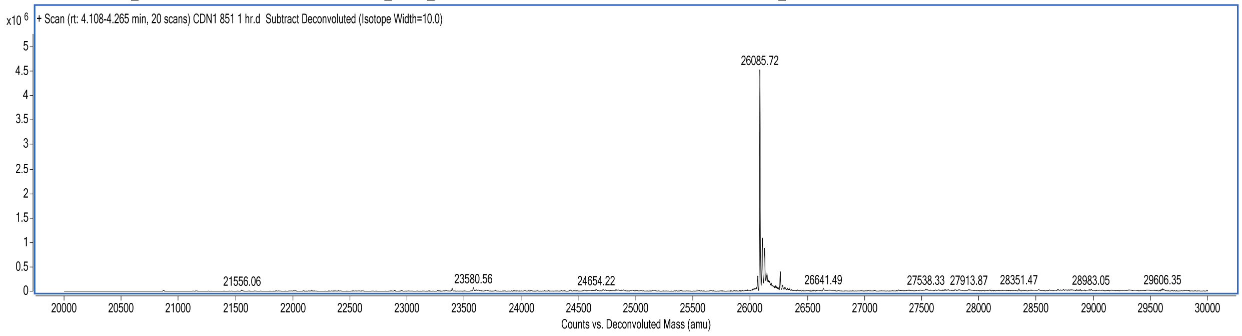


The mass spectrum of DCN1 apo-protein incubated with compound **5** for 3 h:

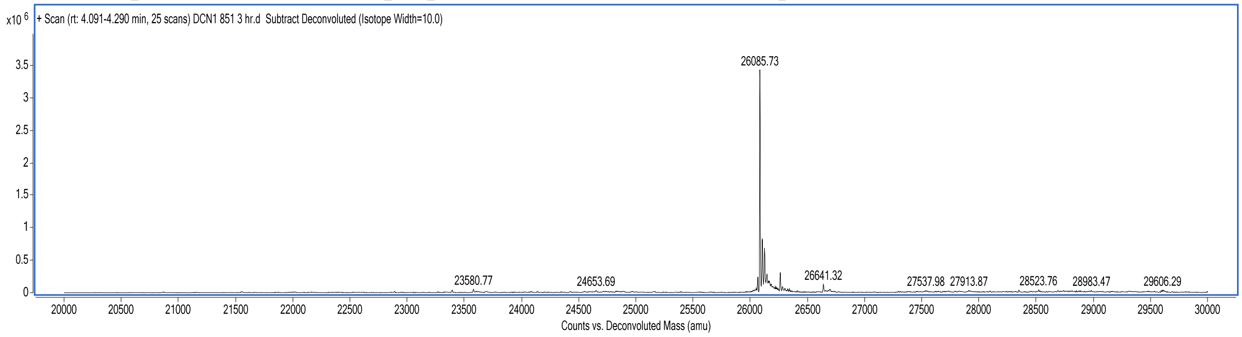


The mass spectrum of DCN1 apo-protein incubated with compound **5** for 12 h:

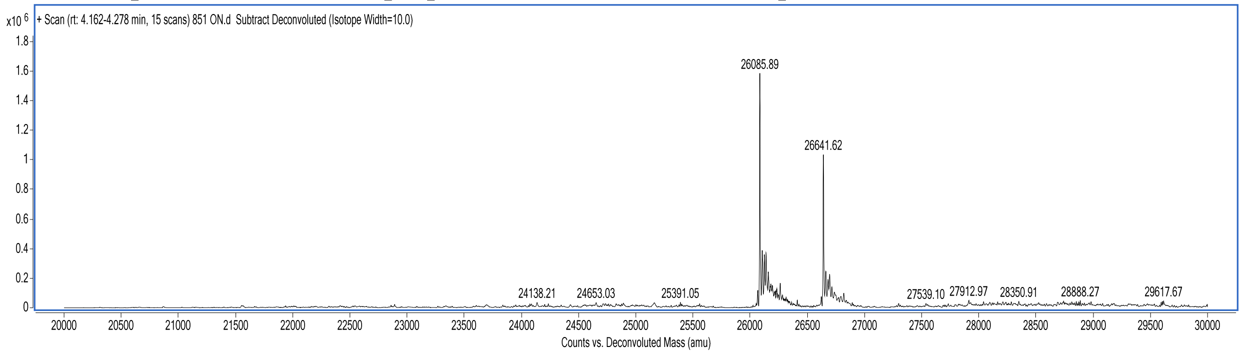


**Supplementary Figure 6. The mass-spectrometric analyses of DCN1 apo-protein incubated with compound 5 for indicated time.**

The mass spectrum of DCN1 apo-protein incubated with DI-1548 for 10 min:

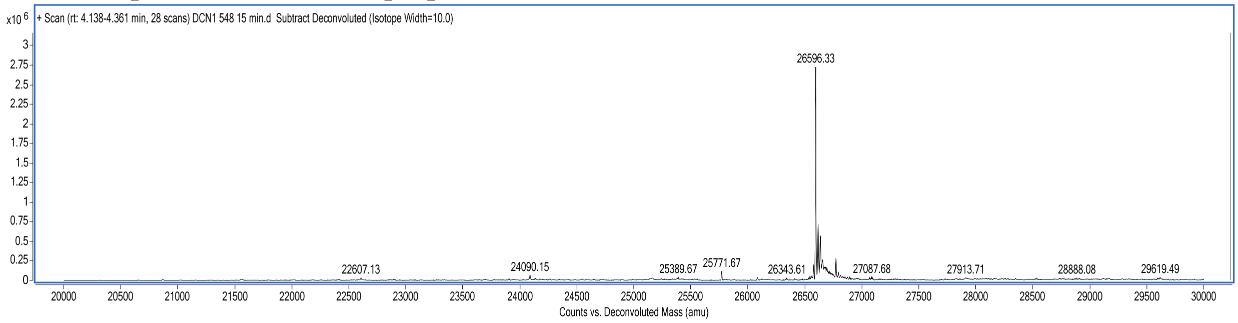


The mass spectrum of DCN1 apo-protein incubated with DI-1548 for 1 h:

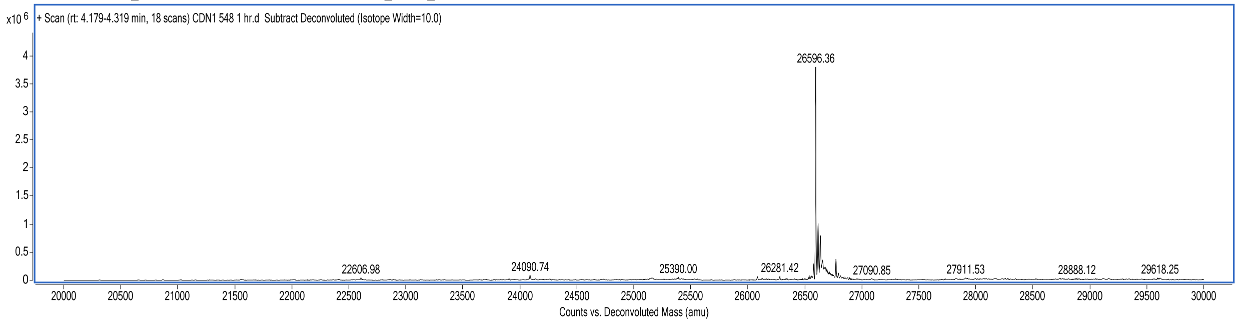


The mass spectrum of DCN1 apo-protein incubated with DI-1548 for 3 h:

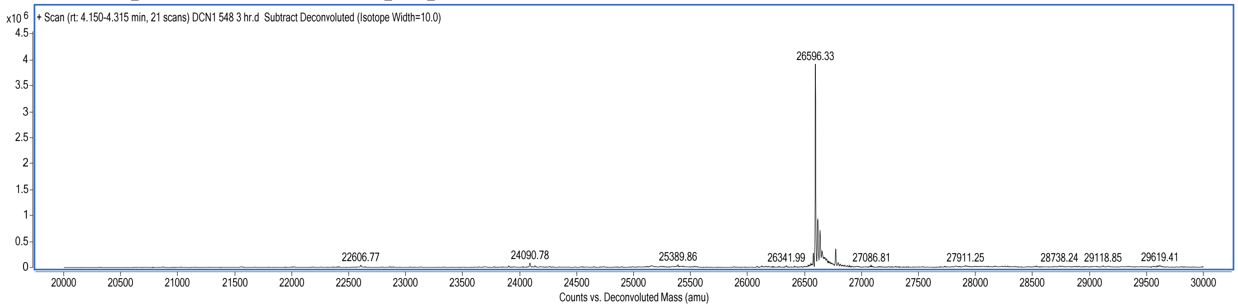


**Supplementary Figure 7. The mass-spectrometric analyses of DCN1 apo-protein incubated with DI-1548 (6) for indicated time.**

The mass spectrum of DCN1 apo-protein incubated with DI-1859 for 10 min:

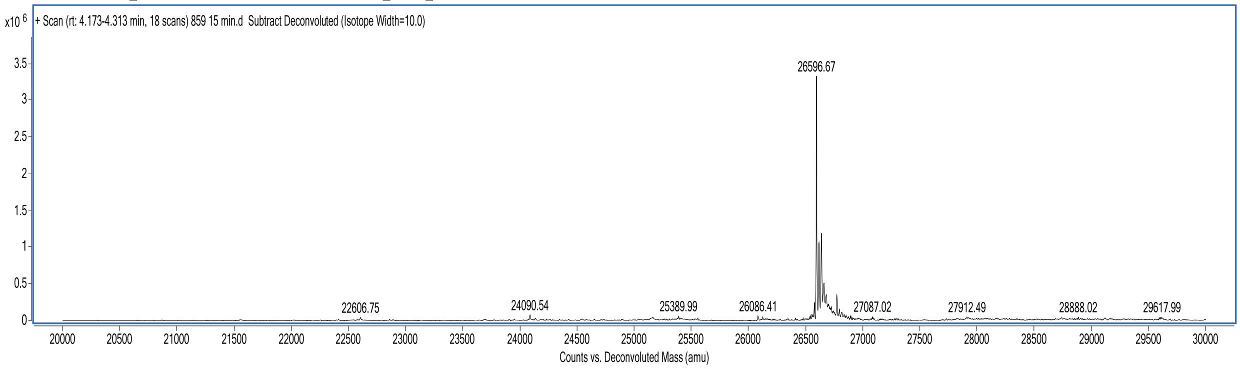


The mass spectrum of DCN1 apo-protein incubated with DI-1859 for 1 h:

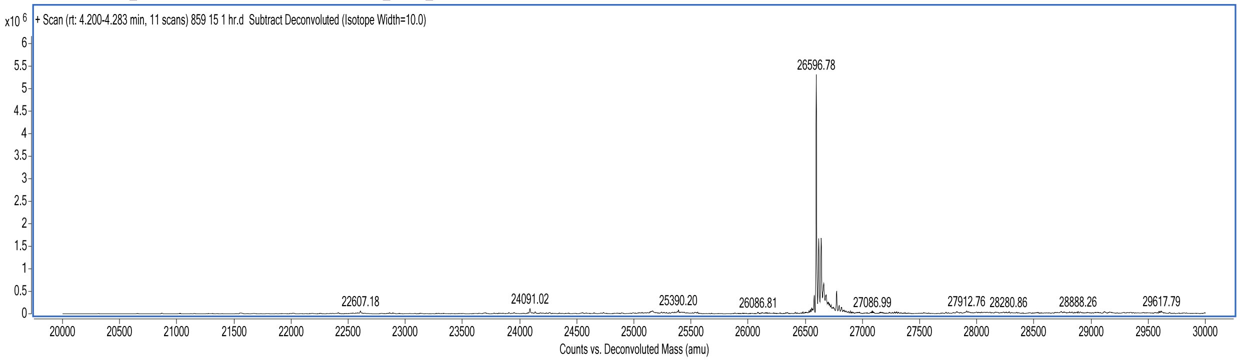


The mass spectrum of DCN1 apo-protein incubated with DI-1859 for 3 h:

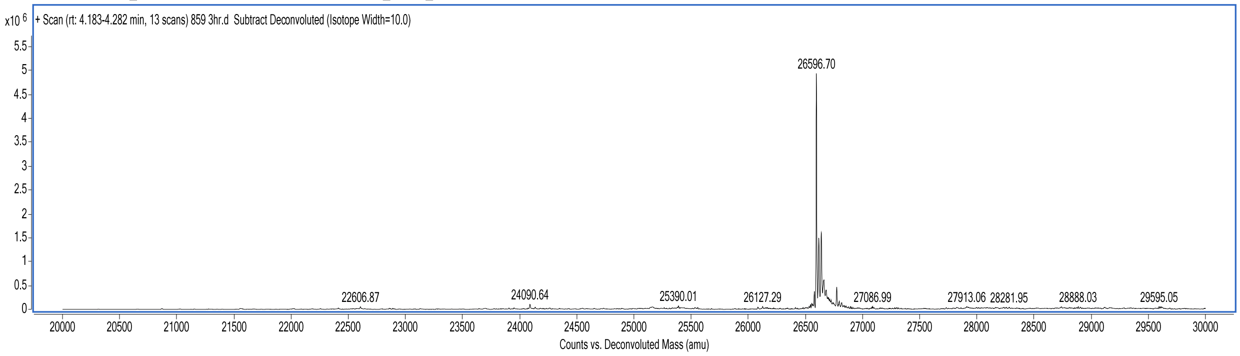


**Supplementary Figure 8. The mass-spectrometric analyses of DCN1 apo-protein incubated with DI-1859 (7) for indicated time.**

The mass spectrum of DCN1 apo-protein incubated with compound **8** for 10 min:

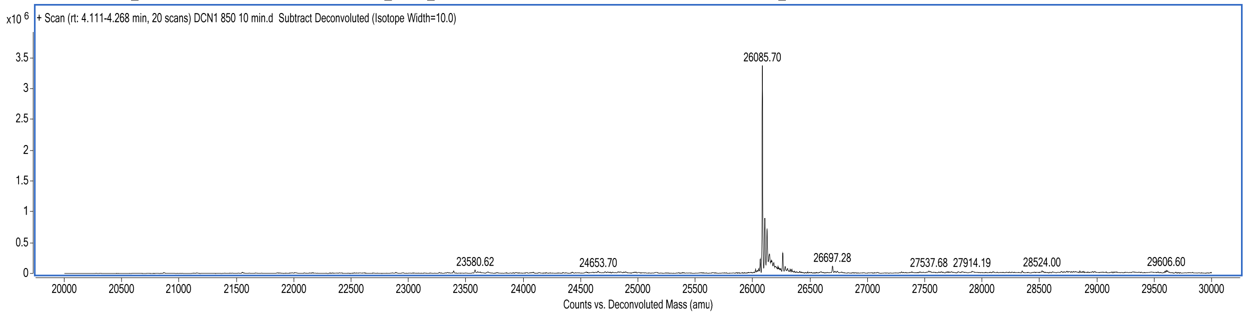


The mass spectrum of DCN1 apo-protein incubated with compound **8** for 1 h:

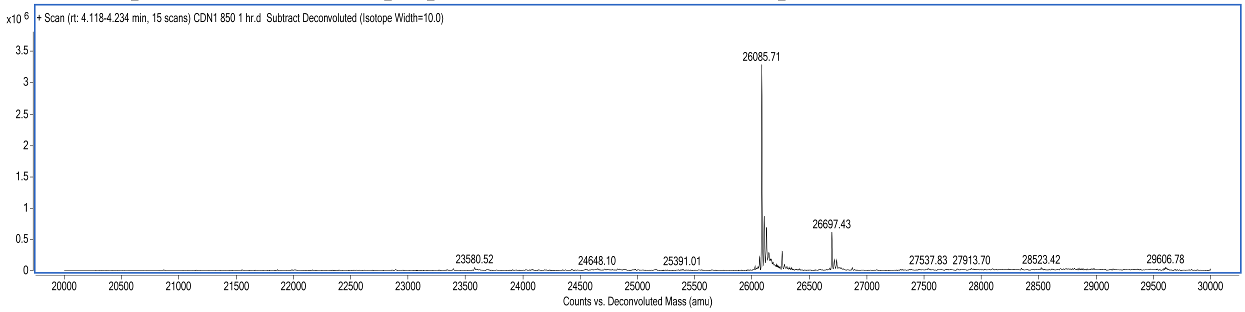


The mass spectrum of DCN1 apo-protein incubated with compound **8** for 3 h:

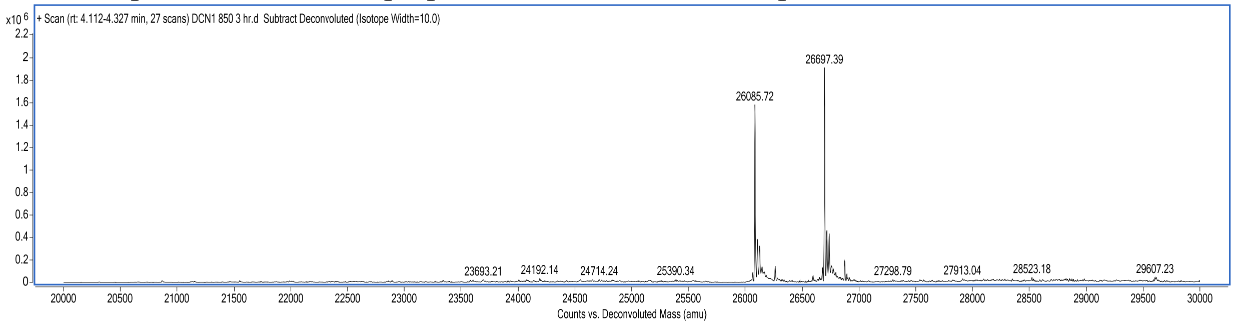


The mass spectrum of DCN1 apo-protein incubated with compound **8** for 12 h:

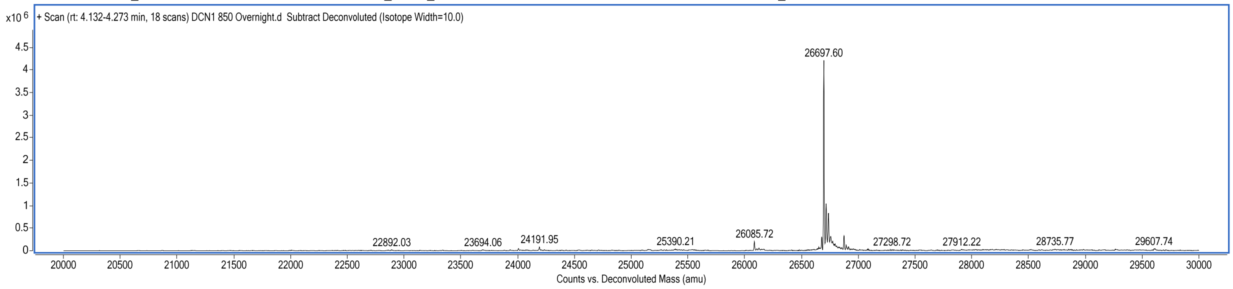


**Supplementary Figure 9. The mass-spectrometric analyses of DCN1 apo-protein incubated with compound 8 for indicated time.**

The mass spectrum of DCN1 apo-protein incubated with compound **9** for 10 min:

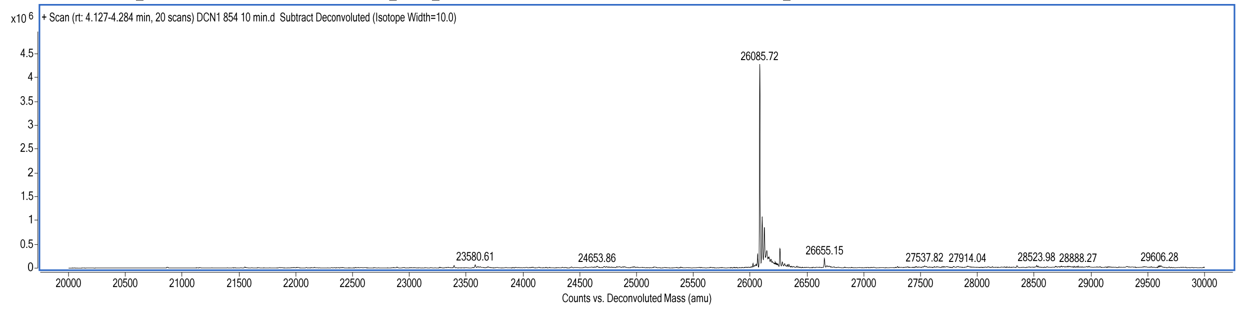


The mass spectrum of DCN1 apo-protein incubated with compound **9** for 1 h:

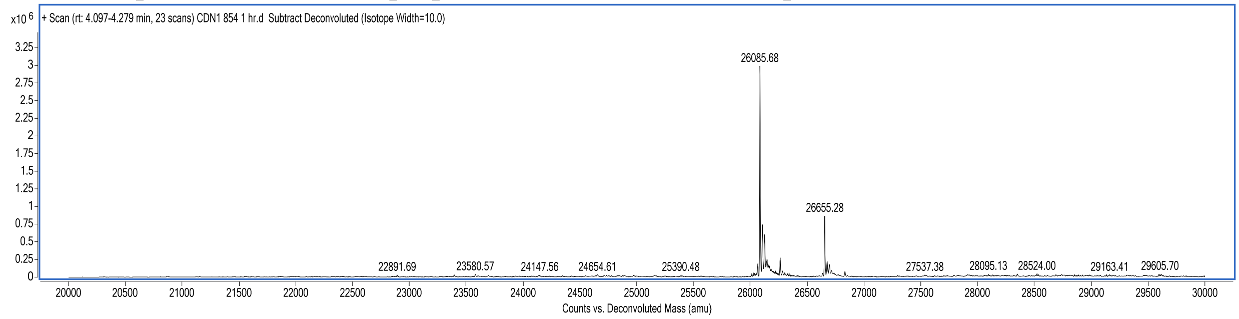


The mass spectrum of DCN1 apo-protein incubated with compound **9** for 3 h:

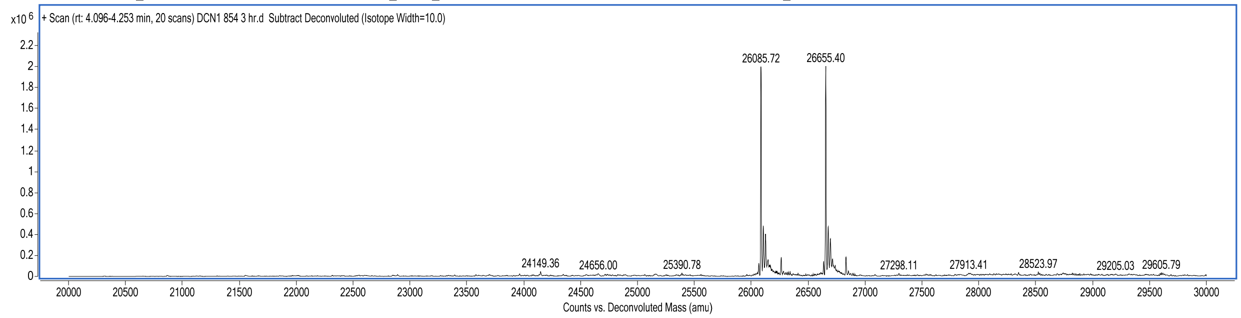


The mass spectrum of DCN1 apo-protein incubated with compound **9** for 12 h:

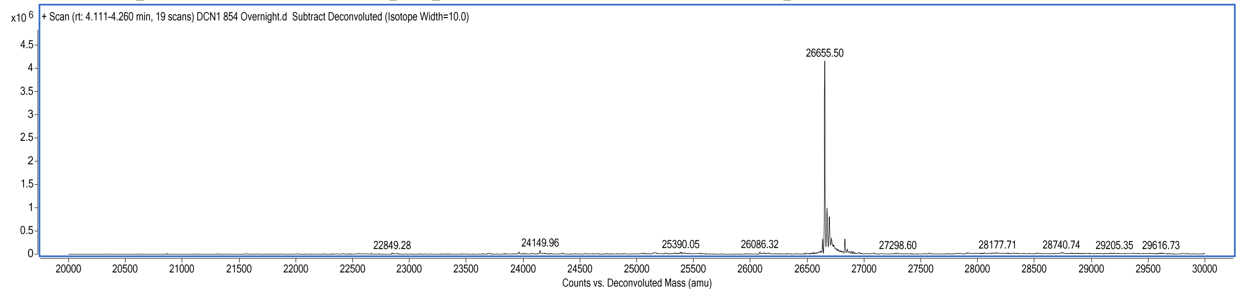


**Supplementary Figure 10. The mass-spectrometric analyses of DCN1 apo-protein incubated with compound 9 for indicated time.**

The mass spectrum of DCN1 apo-protein incubated with compound **10** for 10 min:

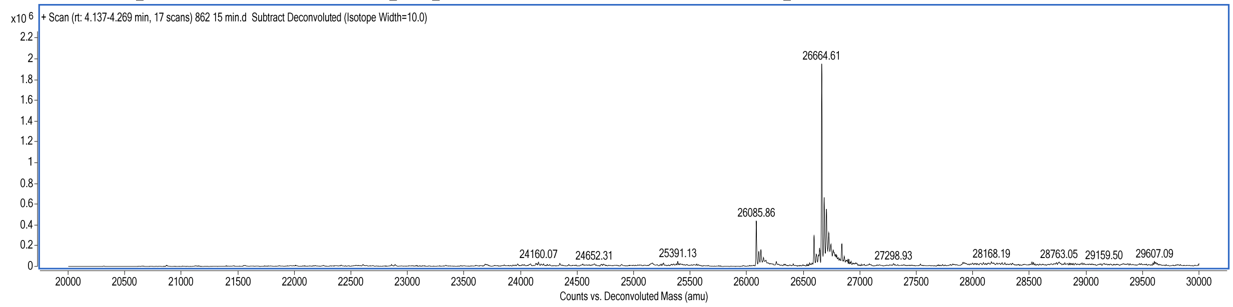


The mass spectrum of DCN1 apo-protein incubated with compound **10** for 1 h:

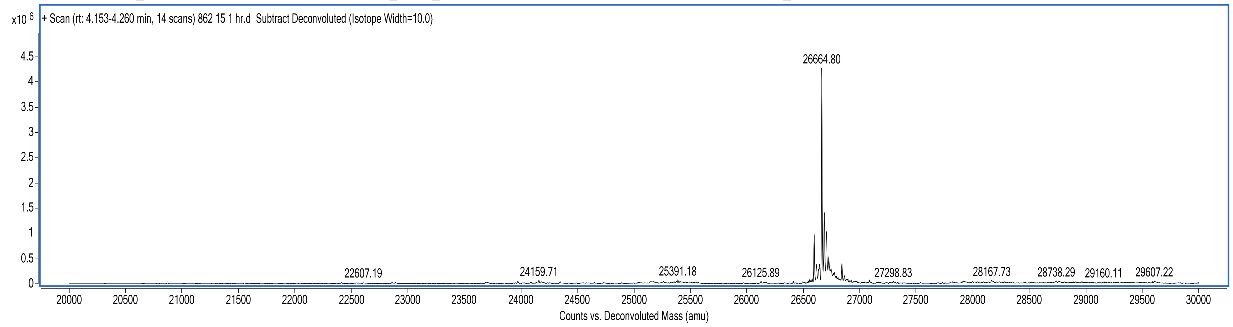


The mass spectrum of DCN1 apo-protein incubated with compound **10** for 3 h:

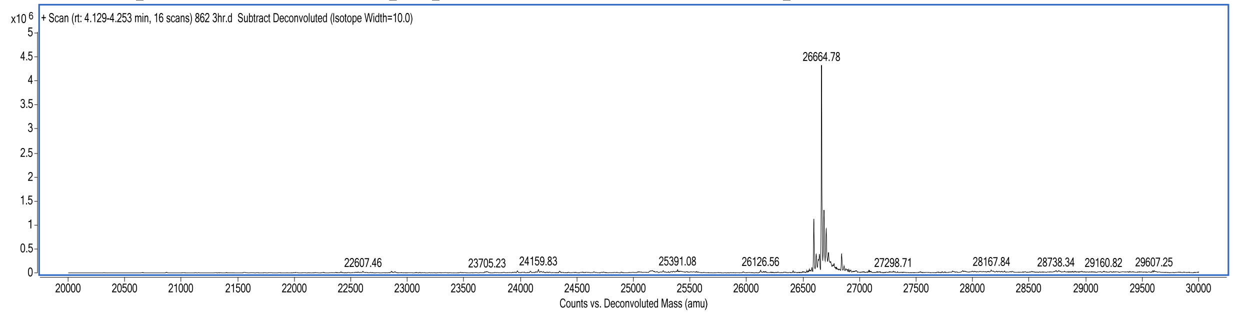


The mass spectrum of DCN1 apo-protein incubated with compound **10** for 12 h:

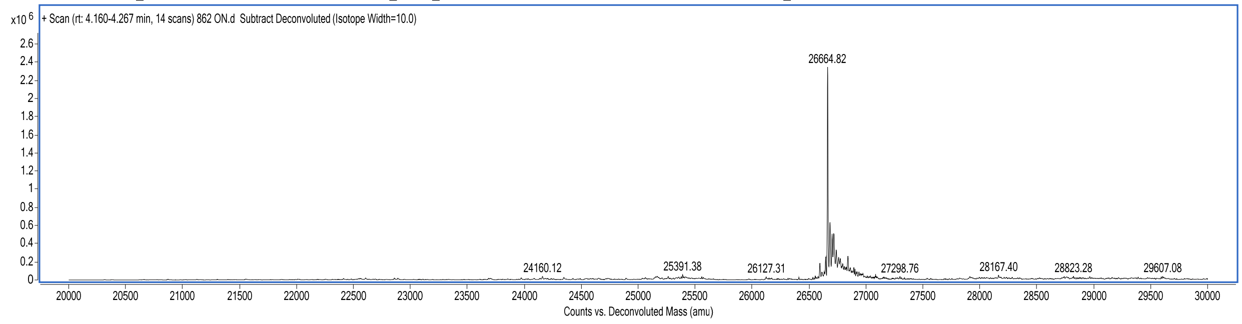


**Supplementary Figure 11. The mass-spectrometric analyses of DCN1 apo-protein incubated with compound 10 for indicated time.**

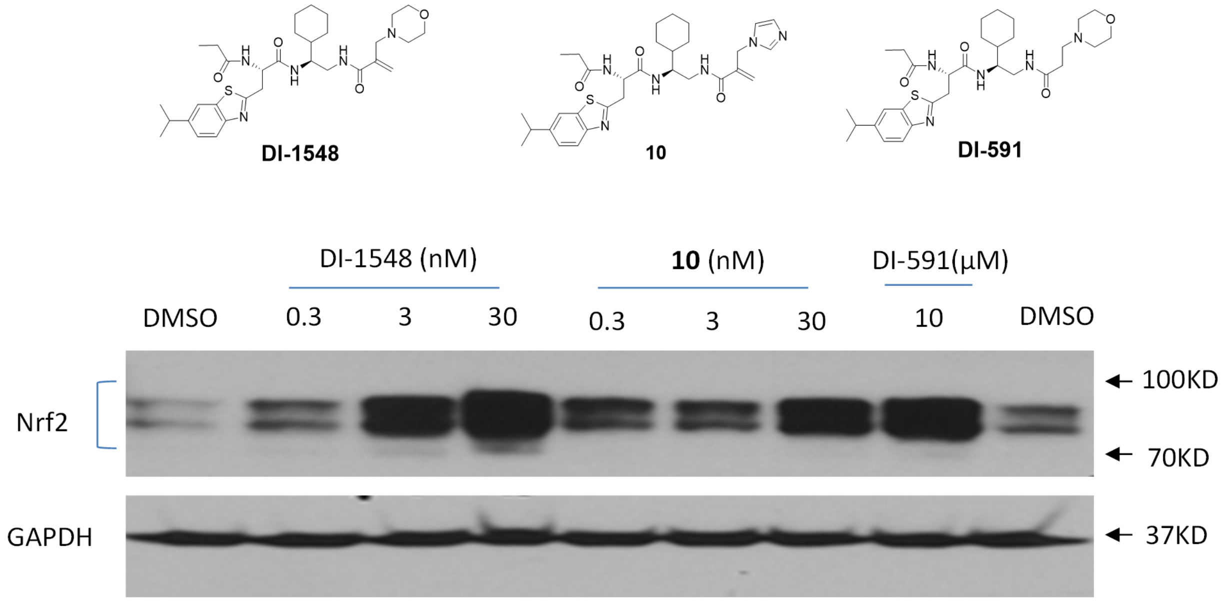


**Supplementary Figure 12. U2OS cell line was treated as indicated for 24 h, the expression of Nrf2 was examined by western blotting.** GAPDH was used as a loading control. Representative images of two independent experiments are shown.

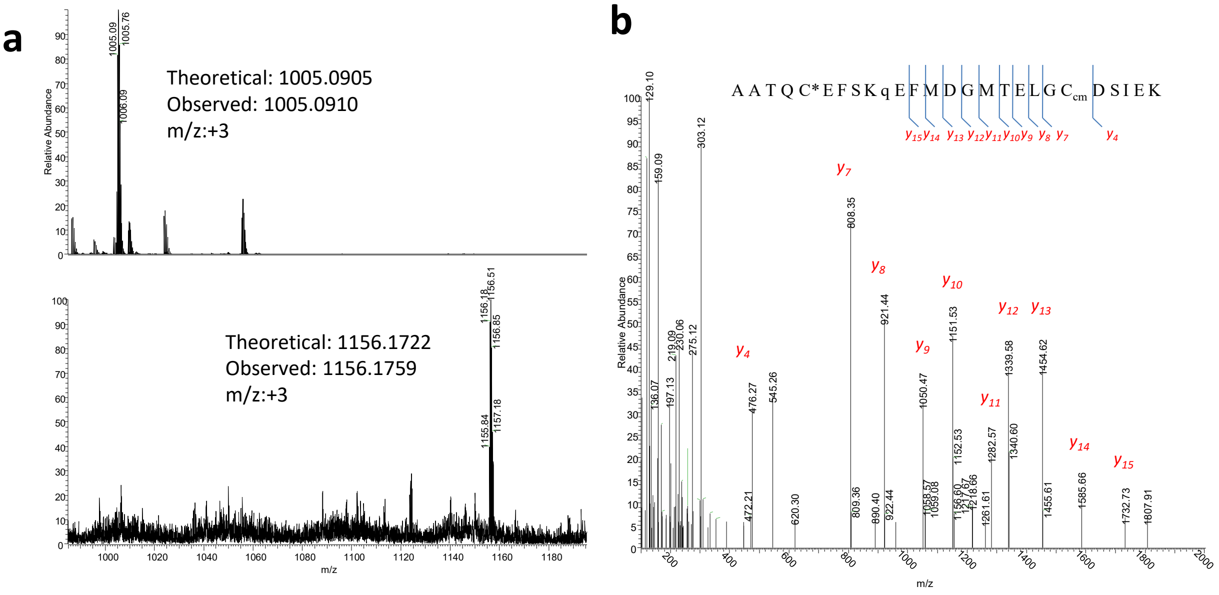


**Supplementary Figure 13. Mass spectrometric analysis of the modification of DCN1 by DI-1548. a** The MS1 spectra (High-resolution LC-tandem Mass spectrometry, Q Exactive HF, Thermoscientific) of tryptic digested peptide (+3 charged) from unmodified and modified DCN1. The observed peak 1156.1759 and 1005.0910 Da were assigned to peptic peptide 111-136, in which the upper panel corresponds to the peptide where both cysteines are carbamidomethylated and the lower panel shows the same peptide modified with one molecule of DI-1548 and carbamidomethyl group. **b** MS/MS spectrum of peptic peptide $_{111}$AATQC$_{DI}$EFSKqEFMDGMTELGC$_{cm}$DSIEK$_{136}$ which is covalently modified by DI-1548 at Cys115. The observed y-ions are indicated. C$_{DI}$= Cysteine modified with DI-1548; C$_{cm}$= Carbamidomethyl Cysteine; q= deamidated glutamine. Source data are provided as a Source Data file.

The mass spectrum of DCN1 apo-protein:

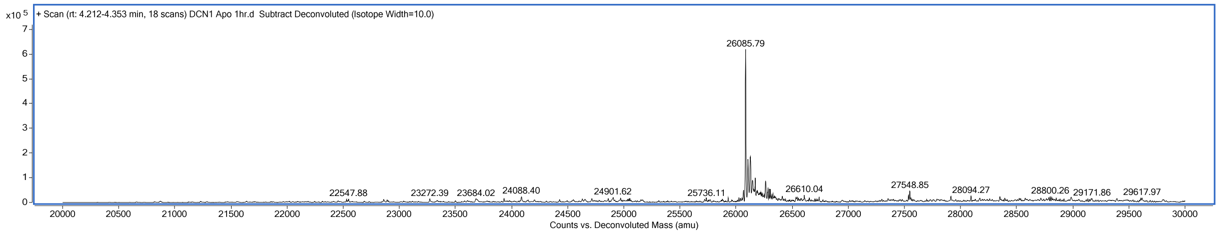


The mass spectrum of DCN1 apo-protein incubated with DI-1548 for 10 min:

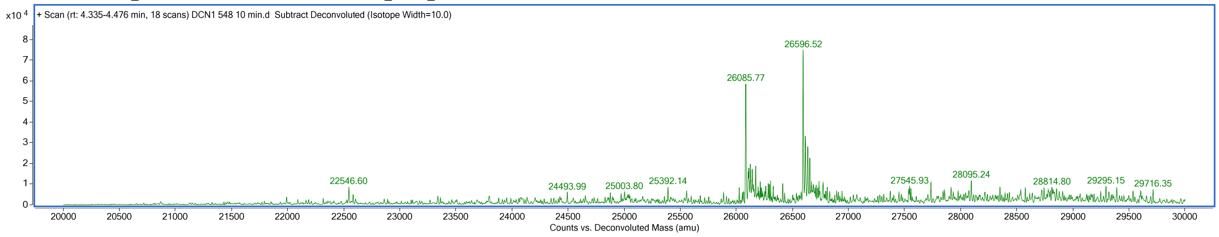


The mass spectrum of DCN1 apo-protein incubated with DI-1548 for 1 h:

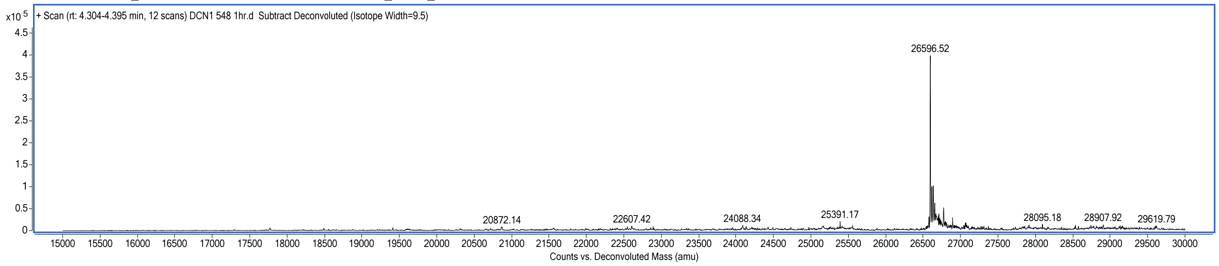


The mass spectrum of DCN1 apo-protein incubated with DI-1548 for 3 h:

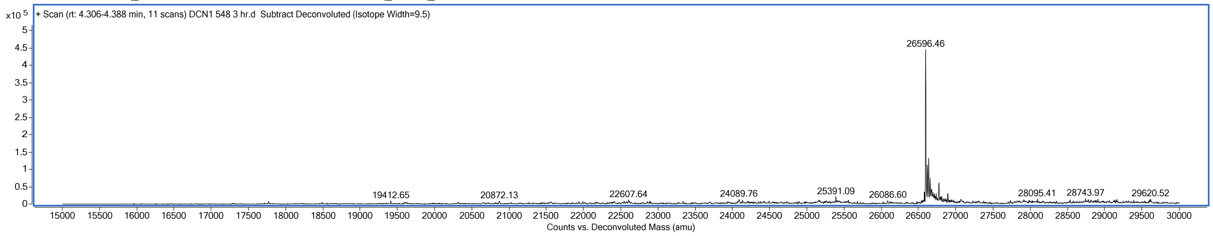


**Supplementary Figure 14. The mass-spectrometric analysis of DCN1 apo-protein and the DCN1 apo-protein incubated with DI-1548 (6) for indicated time.**

The mass spectrum of DCN1 apo-protein incubated with DI-1548DD for 10 min:

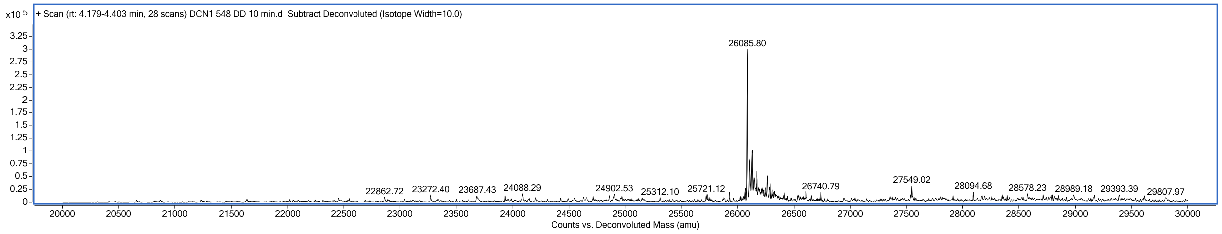


The mass spectrum of DCN1 apo-protein incubated with DI-1548DD for 1 h:

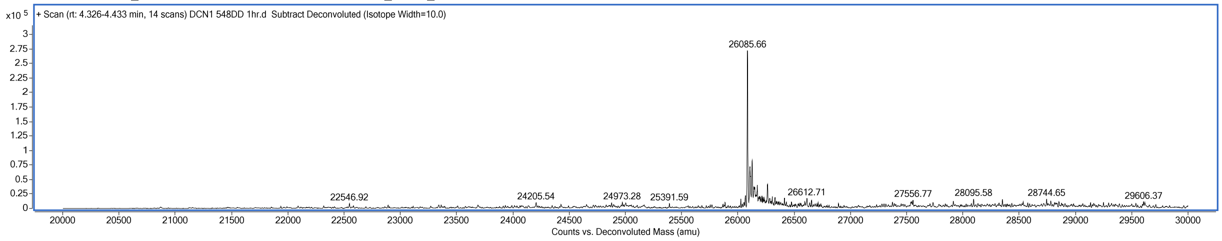


The mass spectrum of DCN1 apo-protein incubated with DI-1548DD for 3 h:

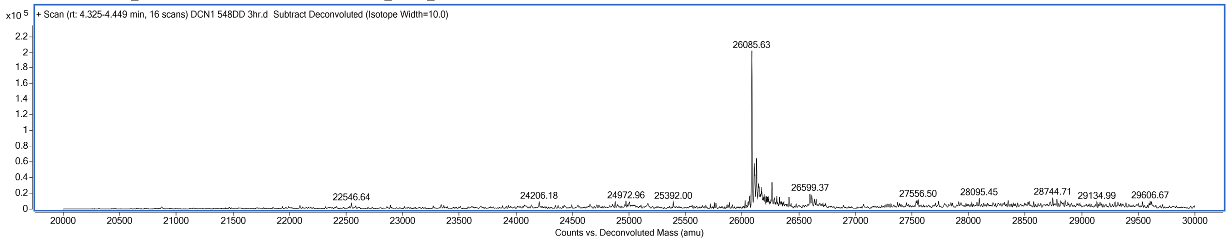


**Supplementary Figure 15. The mass-spectrometric analysis of the DCN1 apo-protein incubated with DI-1548DD (11) for indicated time.**

The mass spectrum of DCN3 apo-protein

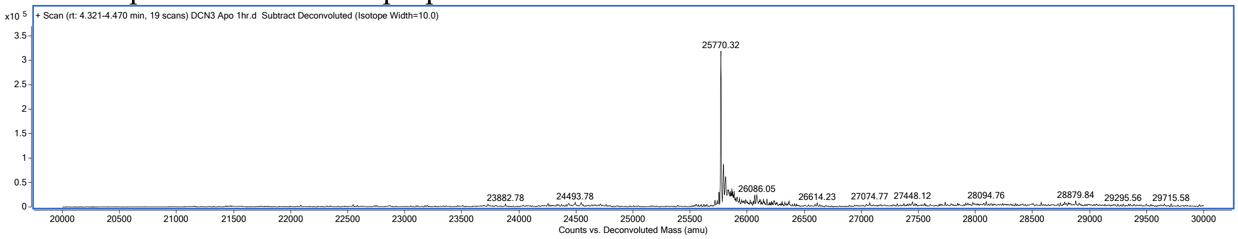


The mass spectrum of DCN3 apo-protein incubated with DI-1548 for 1 h.

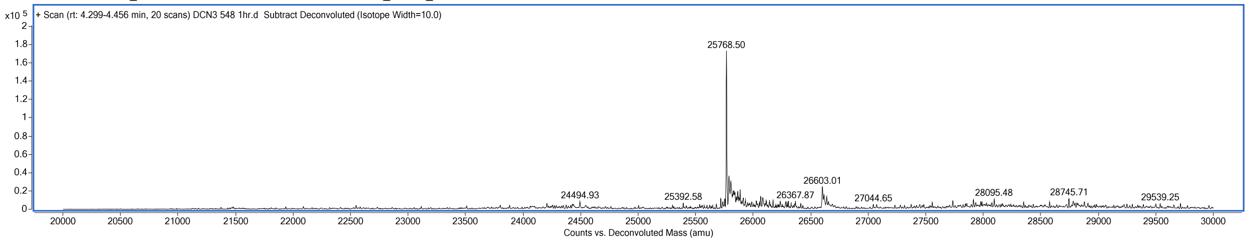


The mass spectrum of DCN3 apo-protein incubated with DI-1548 for 3 h.

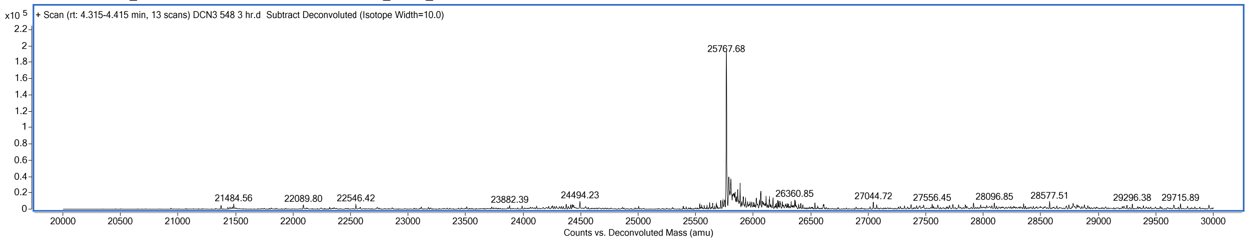


**Supplementary Figure 16. The mass-spectrometric analysis of DCN3 apo-protein and the DCN1 apo-protein incubated with DI-1548 (6) for indicated time.**

The mass spectrum of DCN3 apo-protein incubated with DI-1859 for 1 h.

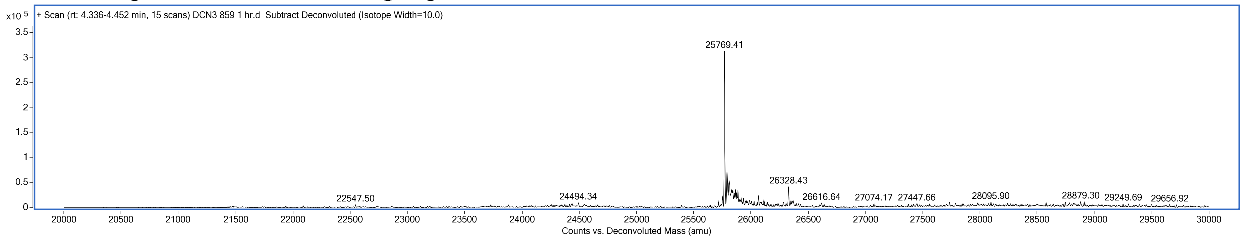


The mass spectrum of DCN3 apo-protein incubated with DI-1859 for 3 h.

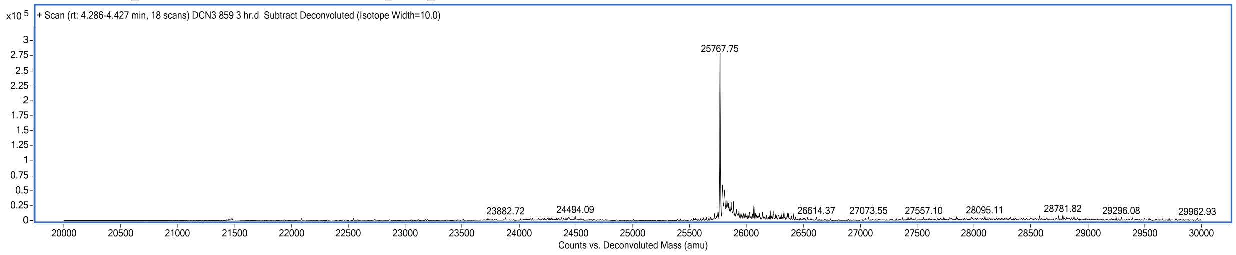


**Supplementary Figure 17. The mass-spectrometric analysis of DCN3 apo-protein and the DCN1 apo-protein incubated with DI-1859 (7) for indicated time.**

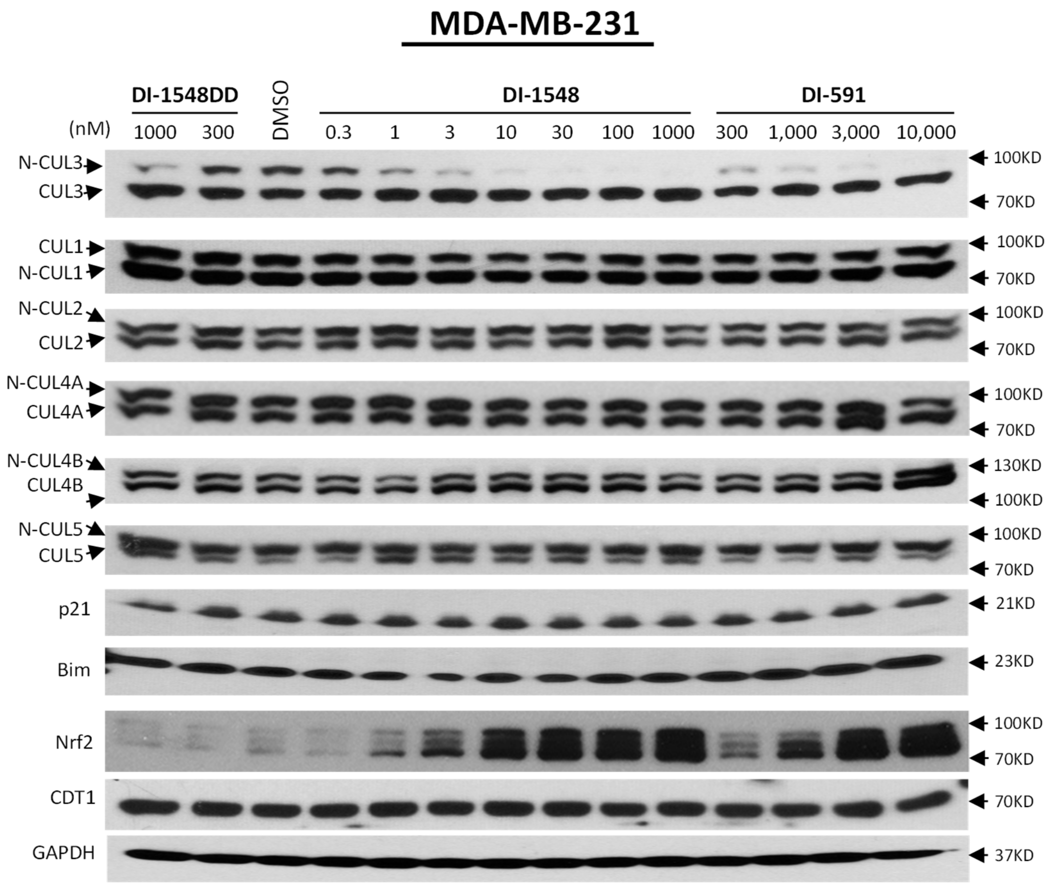


**Supplementary Figure 18. DI-1548 selectively inhibited Cullin3 neddylation in breast cancer MDA-MB-231 cell lines.** MDA-MB-231 cell line was treated by dose-ranges of compound DI-1548, DI-1548DD, or DI-591 for 24 h. The protein levels of neddylated-cullin3, 1, 2, 4A, 4B and 5 (N-Cul3, 1, 2, 4A, 4B and 5) and un-neddylated-cullin3, 1, 2, 4A, 4B and 5 (Cul3, 1, 2, 4A, 4B and 5), as well as the substrates of cullins-mediated CRLs, including p21, Bim, Nrf2 and CDT1 were examined by western blotting analysis. GAPDH was used as a loading control. Representative images of two independent experiments are shown.

## KYSE70

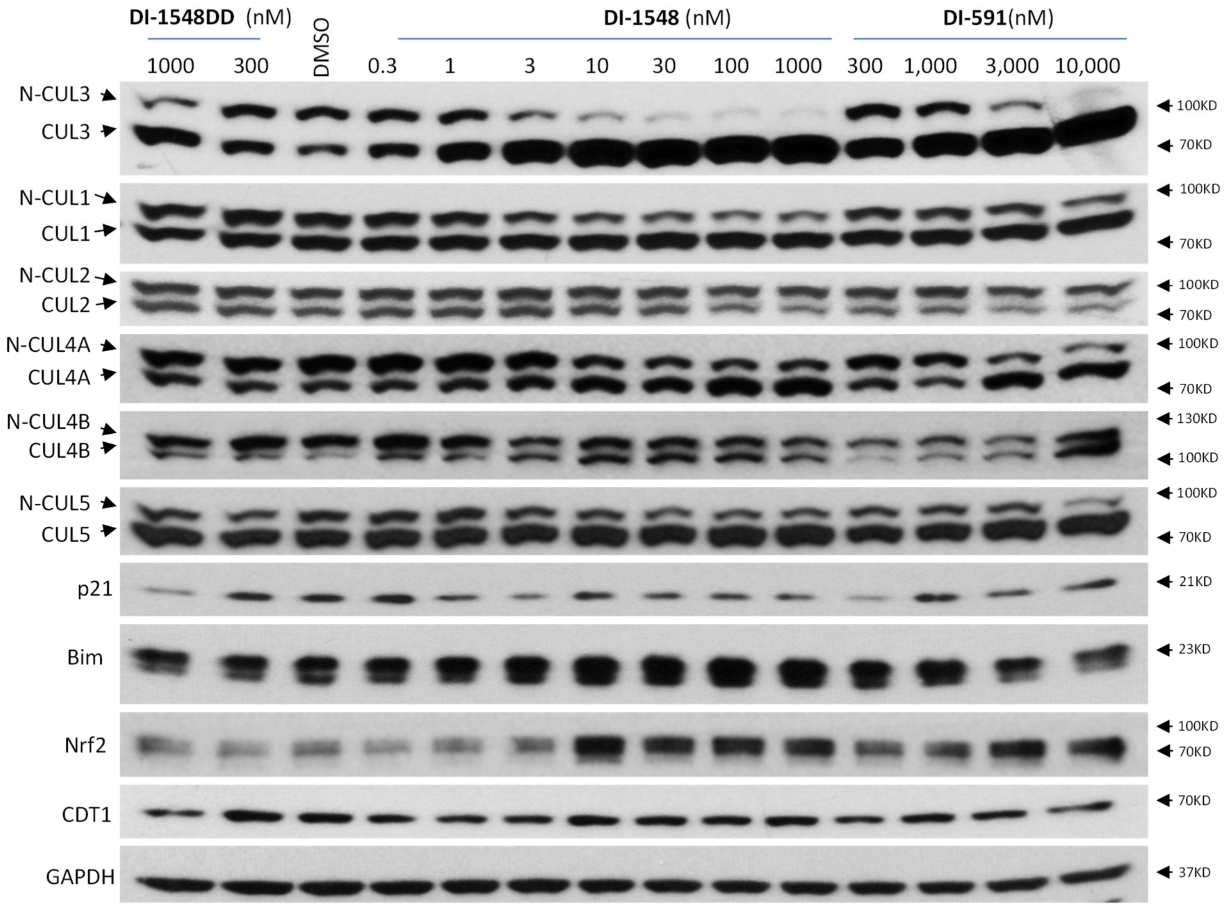


**Supplementary Figure 19. DI-1548 selectively inhibited Cullin3 neddylation in esophageal cancer KYSE70 cells.** Esophageal cancer KYSE70 cell line was treated by dose-ranges of compound DI-1548, DI-1548DD, or DI-591 for 24 h. The protein levels of neddylated-cullin3, 1, 2, 4A, 4B and 5 (N-Cul3, 1, 2, 4A, 4B and 5) and un-neddylated-cullin3, 1, 2, 4A, 4B and 5 (Cul3, 1, 2, 4A, 4B and 5), as well as the substrates of cullins-mediated CRLs, including p21, Bim, Nrf2 and CDT1 were examined by western blotting analysis. GAPDH was used as a loading control. Representative images of two independent experiments are shown.

**HCC116**

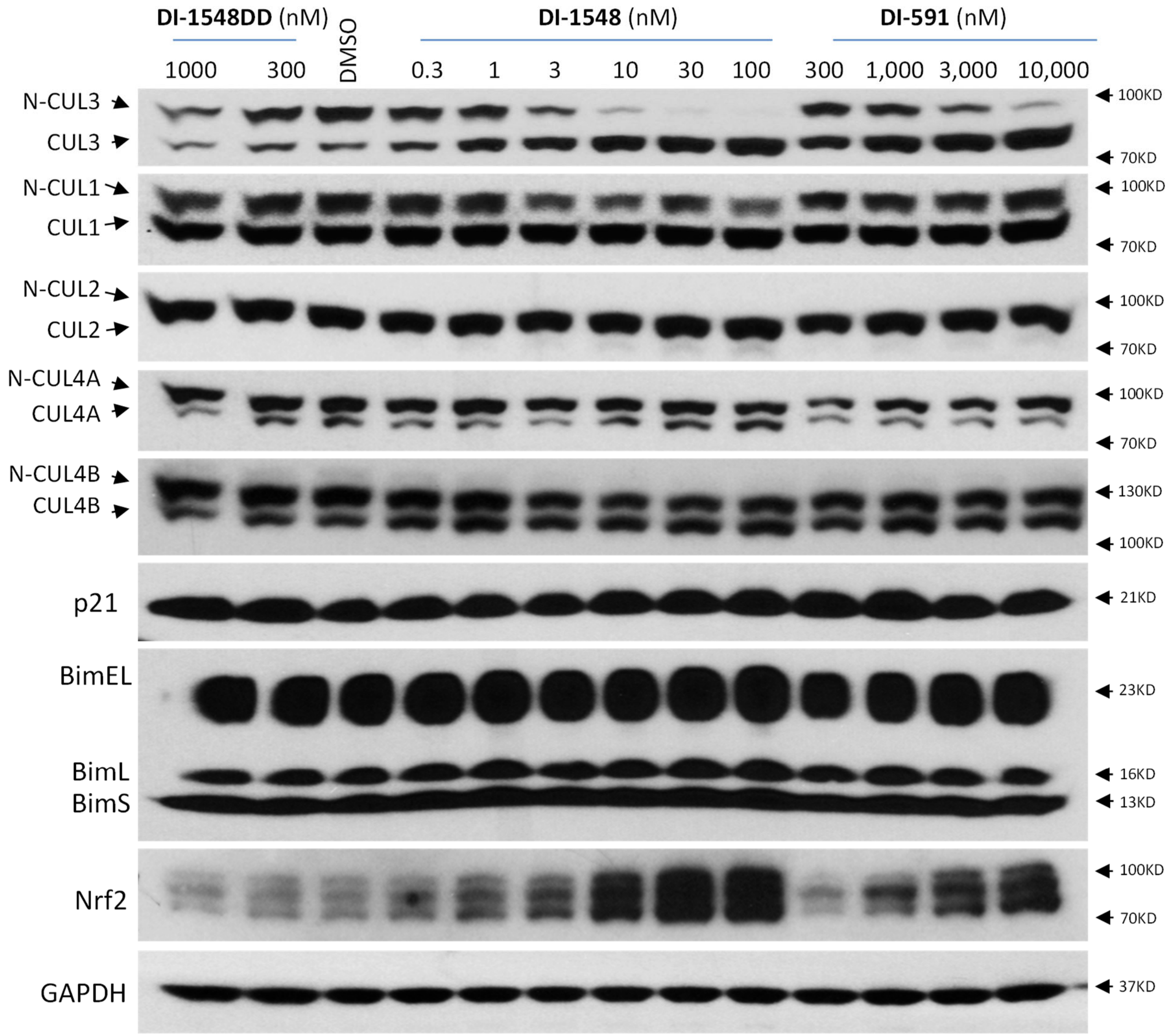


**Supplementary Figure 20. DI-1548 selectively inhibited Cullin3 neddylation in in colon cancer HCC116 cell line.** Colon cancer HCC116 cell line was treated by dose-ranges of compound DI-1548, DI-1548DD, or DI-591 for 24 h. The protein levels of neddylated-cullin3, 1, 2, 4A, 4B and 5 (N-Cul3, 1, 2, 4A, 4B and 5) and un-neddylated-cullin3, 1, 2, 4A, 4B and 5 (Cul3, 1, 2, 4A, 4B and 5), as well as the substrates of cullins-mediated CRLs, including p21, Bim, Nrf2 and CDT1 were examined by western blotting analysis. GAPDH was used as a loading control. Representative images of two independent experiments are shown.

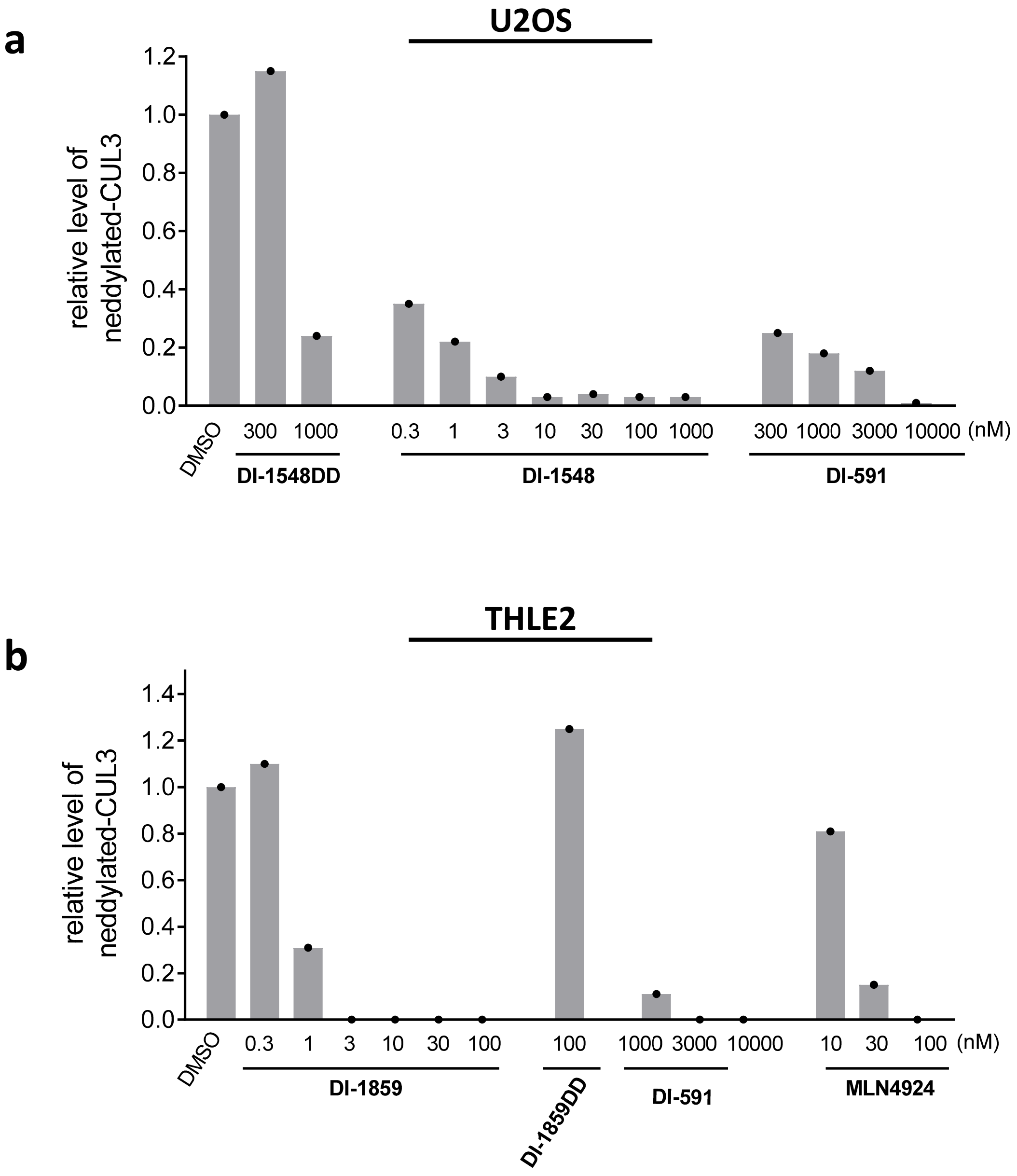


**Supplementary Figure 21. Densitometry analysis of the neddylated-Cul3 in Figure 6a (a) and 6b (b).** Densitometry analysis was carried out by ImageJ software and the relative numbers were plotted.

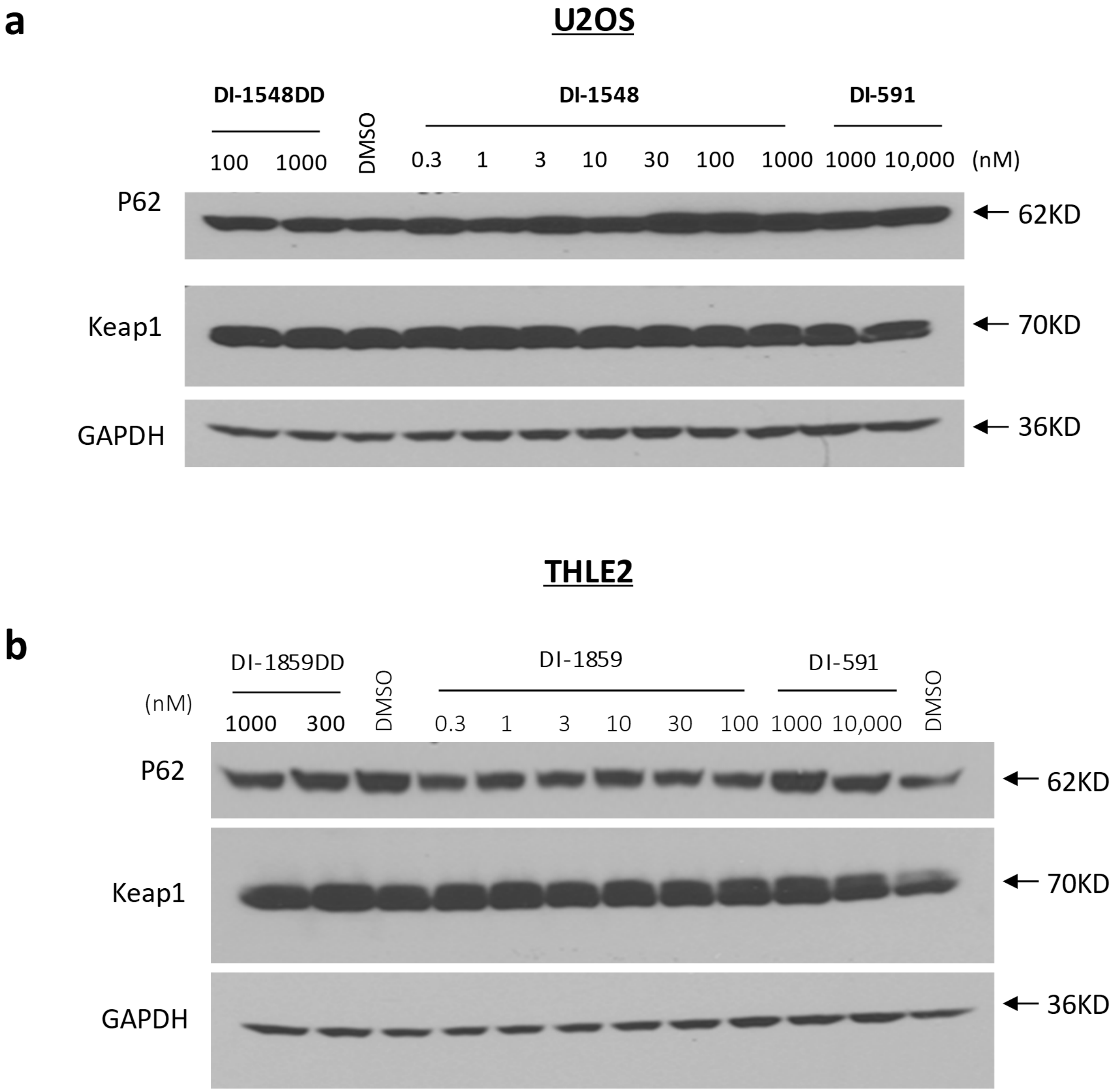


**Supplementary Figure 22. Western blotting analysis of Keap1 and p62 in U2OS cells (a) and THLE2 cells (b) treated with DI-1548, DI-1548DD and DI-591.** The cells were treated with compounds as indicated for 6 h. Treated cells were harvested and lysed with RIPA buffer. The expression of P62 and Keap1 were examined by western blotting analysis. Antibody information: Keap1 (cst, #8047), P62 (cst,#5114). GAPDH was used as a loading control. Representative images of two independent experiments are shown.

**THLE2**

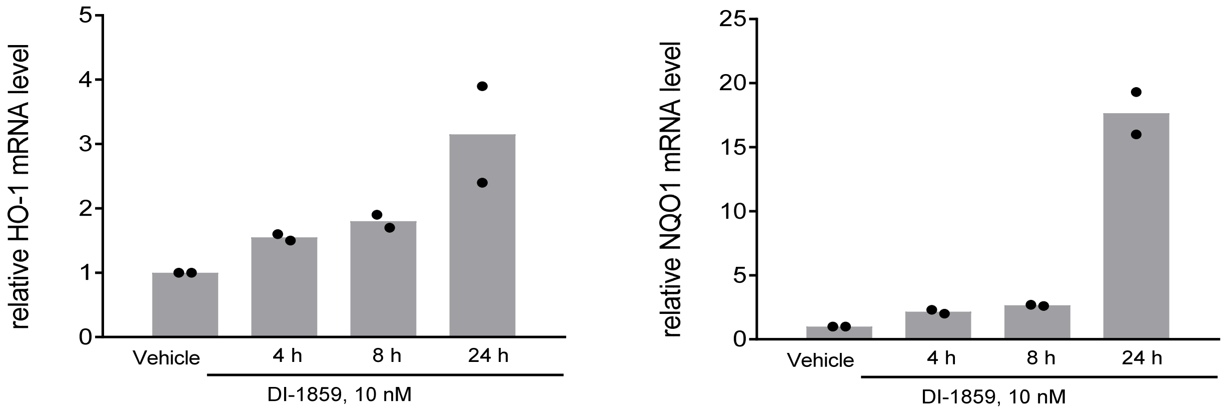


**Supplementary Figure 23. DI-1859 increases the mRNA level of NRF2 targeted genes *NQO1* and *HO-1*.** THLE2 cells were treated with DI-1859 at 10 nM for 4, 8 and 24 h. Treated cells were harvested and the mRNA level of *HO-1* and *NQO1* were examined by RT-PCR assay. The probes for *HO-1* (Hs01110250_m1) and *NQO1* (Hs01045993_g1) were purchased from ThermoFisher Scientific (Ann Arbor, MI). Data are presented as mean values with n = 2 independent biological replicates

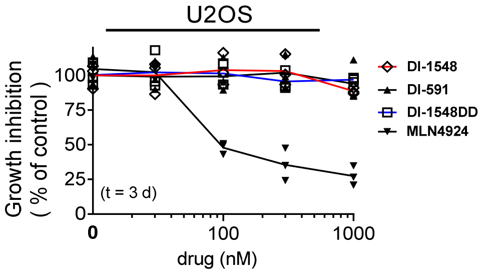


| | DI-1548DD | DI-1548DD | DI-591 (100 nM) | MLN4924 |
|---|---|---|---|---|
| EC50 | 6411 | 12736 | 48210 | 208.6 |

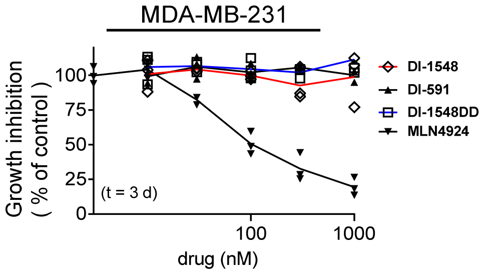


| | DI-1548 | DI-1548DD | DI-591 | MLN4924 |
|---|---|---|---|---|
| EC50 | 3010 | 4910 | 5396 | 154.2 |

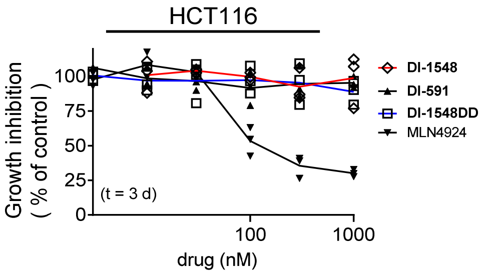


| | DI-1548 | DI-1548DD | DI-591 | MLN4924 |
|---|---|---|---|---|
| EC50 | 3010 | 5757 | 51281 | 294.5 |

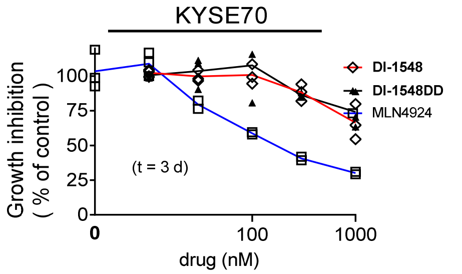


| | DI-1548 | MLN4924 | DI-1548DD |
|---|---|---|---|
| EC50 | 3056 | 265.7 | 9372 |

**Supplementary Figure 24. Cell growth inhibitory activity of compounds DI-1548, DI-1548DD, DI-591 and MLN4924 in a panel of 4 cell lines.** Cell lines were treated by DI-1548, DI-1548DD, DI-591 and MLN4924 for 72 h. Cell viability was determined with a WST assay. Data are presented as mean values ± s.d. with n = 3 independent biological replicates.

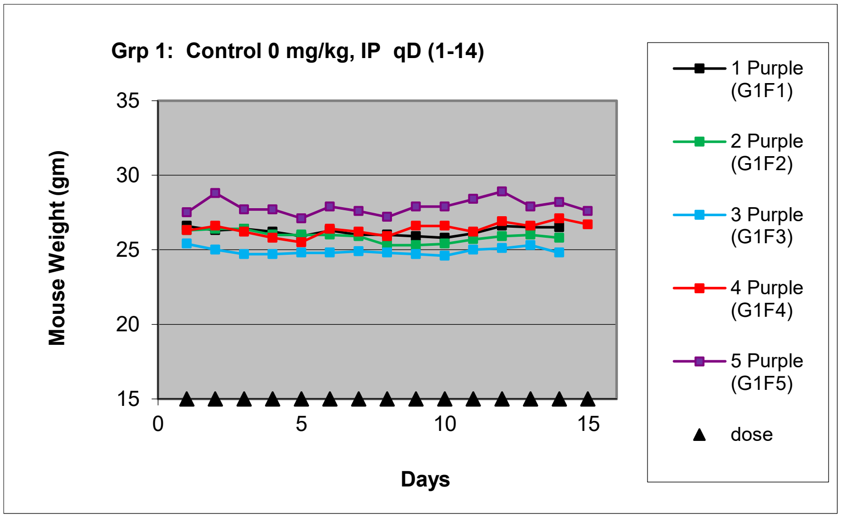


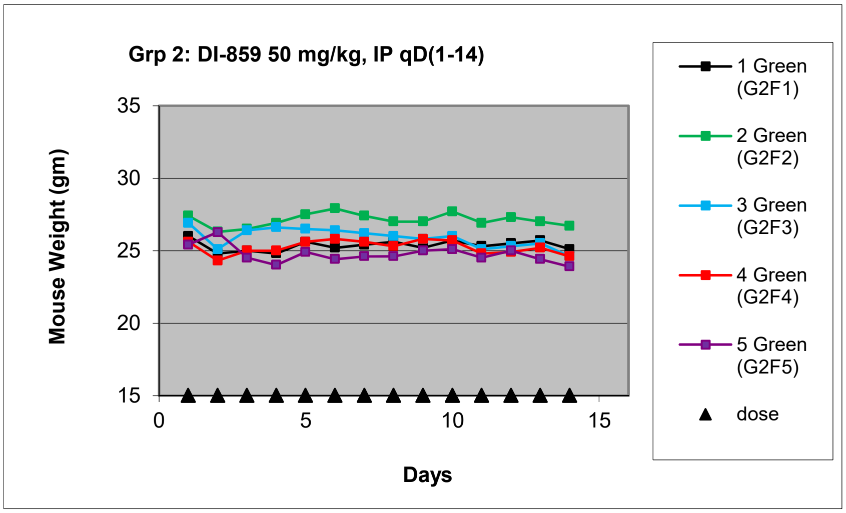


**Supplementary Figure 25. The mouse body weight of the male C57BL/6J mice treated (IP) with vehicle (Group 1, n = 5) or DI-1859 at 50 mg/kg (Group 2, n = 5).**

**Supplementary Table 1:** Crystallography Data Collection and Refinement Statistics.

| **Data Collection** | **DCN1-DI-1548** | **8** | **9** | **DCN1-DI-1859** | **10** | **4** |
|---|---|---|---|---|---|---|
| PDB ID | 6XOL | 6XOM | 6XON | 6XOO | 6XOP | 6XOQ |
| Space Group | $P2_12_12_1$ | $P2_12_12_1$ | $P2_12_12_1$ | $P2_12_12_1$ | $P2_12_12_1$ | $P2_12_12_1$ |
| Unit Cell (Å) | a = 33.910<br>b = 86.420<br>c = 126.020<br>α = β = γ = 90° | a = 33.721 b = 86.698 c = 126.082<br>α = β = γ = 90° | a = 33.444<br>b = 85.610<br>c = 125.164<br>α = β = γ = 90° | a = 34.020<br>b = 86.865<br>c = 126.554<br>α = β = γ = 90° | a = 33.868<br>b = 86.702<br>c = 126.234<br>α = β = γ = 90° | a = 33.993<br>b = 86.850<br>c = 126.146<br>α = β = γ = 90° |
| Wavelength (Å) | 0.9786 | 0.9786 | 0.9786 | 0.9786 | 0.9786 | 0.9786 |
| Resolution (Å)[1] | 50-2.39<br>(2.43-2.39) | 50-2.10<br>(2.14-2.10) | 50-2.70<br>(2.75-2.70) | 50-2.06<br>(2.10-2.06) | 50-2.07<br>(2.11-2.07) | 50-2.07<br>(2.11-2.07) |
| Rsym[2] | 0.067 (0.959) | 0.057 (0.565) | 0.081 (0.731) | 0.047 (0.551) | 0.048 (0.356) | 0.053 (0.507) |
| <I/σI>[3] | 12 (2.3) | 13.8 (2.1) | 10.4 (1.8) | 16 (2.2) | 19.3 (4) | 13.3 (2.2) |
| Completeness (%)[4] | 99.9 (99.7) | 100 (100) | 100 (100) | 99.3 (86.3) | 100 (100) | 99.9 (99.8) |
| Redundancy | 6.8 (6.4) | 7.1 (6.3) | 6.7 (6.8) | 7.0 (5.2) | 7.1 (5.9) | 7.0 (5.5) |
| **Refinement** | | | | | | |
| Resolution (Å) | 2.39 | 2.10 | 2.80 | 2.06 | 2.07 | 2.07 |
| R-Factor [5] | 0.212 | 0.195 | 0.221 | 0.197 | 0.198 | 0.202 |
| Rfree [6] | 0.264 | 0.243 | 0.289 | 0.242 | 0.247 | 0.247 |
| Protein atoms | 2815 | 2837 | 2779 | 2818 | 2818 | 2833 |
| Water Molecules | 133 | 239 | 58 | 268 | 292 | 261 |
| Ligands | 1 | 1 | 1 | 1 | 1 | 1 |
| Refined Ligand Occupancy | 1.0 | Core atoms: 1.0<br>Methylmorpholine atoms: 0.5 | 1.0 | 1.0 | 1.0 | Core atoms: 1.0<br>Allymorpholine atoms :0.0 |
| B values (Å$^2$) | | | | | | |
| Overall | 42.3 | 31.5 | 42.2 | 32.2 | 30.95 | 31.0 |
| Protein atoms | 42.4 | 30.9 | 42.5 | 31.8 | 30.34 | 30.5 |
| Ligand atoms | 41.7 | 35.5 | 42.7 | 29.6 | 34.5 | 32.6 |
| Waters | 40.9 | 35.3 | 31.3 | 39.4 | 35.8 | 35.9 |
| Unique Reflections | 15028 | 22377 | 9324 | 23686 | 23490 | 23370 |
| R.m.s.d.[7] | | | | | | |
| Bonds | 0.009 | 0.01 | 0.008 | 0.01 | 0.01 | 0.01 |
| Angles | 0.98 | 0.98 | 0.97 | 0.97 | 0.96 | 0.95 |
| MolProbity Score[8] | 1.12 | 0.88 | 1.22 | 1.21 | 1.05 | 0.84 |
| Clash Score[8] | 1.42 | 0.87 | 1.08 | 1.6 | 1.07 | 1.24 |
| RSCC[9] | 0.92 | 0.94 | 0.91 | 0.95 | 0.92 | 0.91 |
| RSR[9] | 0.18 | 0.14 | 0.28 | 0.12 | 0.15 | 0.15 |

[1]Statistics for highest resolution bin of reflections in parentheses.

[2]$R_{sym} = \Sigma_h \Sigma_j \mid I_{hj} - \langle I_h \rangle \mid / \Sigma_h \Sigma_j I_{hj}$, where $I_{hj}$ is the intensity of observation j of reflection h and $\langle I_h \rangle$ is the mean intensity for multiply recorded reflections.

[3]Intensity signal-to-noise ratio.

[4]Completeness of the unique diffraction data.

[5]R-factor = $\Sigma_h \mid IF_oI - IF_cI \mid / \Sigma_h |F_o|$, where $F_o$ and $F_c$ are the observed and calculated structure factor amplitudes for reflection h.

[6]$R_{free}$ is calculated against a 5% random sampling of the reflections that were removed before structure refinement.

[7]Root mean square deviation of bond lengths and bond angles.
[8]Molprobity reference
[9]wwPDB Validation Server.

**Supplementary Table 2A.** Concentrations of DI-1548 in mouse Plasma and liver after the C57BL/6 male mice were treated with a single dose of DI-1548 at 25 mg/kg via intraperitoneal (IP) injection. BLQ: below the limit of quantification.

| DI-1548, 25 mg/kg, IP | | |
|---|---|---|
| **Time point (h)** | **Plasma (ng/mL)** | **Liver (ng/g)** |
| **1** | 1826 ± 319 | 783 ± 336 |
| **3** | 4.5 ± 3.6 | BLQ |
| **6** | BLQ | BLQ |
| **24** | BLQ | BLQ |

**Supplementary Table 2B.** Concentrations of DI-1859 in mouse Plasma and liver after the C57BL/6 male mice were treated with a single dose of DI-1859 at 25 mg/kg via intraperitoneal (IP) injection. BLQ: below the limit of quantification.

| DI-1859, 25 mg/kg, IP | | |
|---|---|---|
| **Time point (h)** | **Plasma (ng/mL)** | **Liver (ng/g)** |
| **1** | 1737 ± 292 | 3282 ± 531 |
| **3** | 28 ± 12 | 30 ± 29 |
| **6** | BLQ | BLQ |
| **24** | BLQ | BLQ |

**Supplementary Table 3. Overview Toxicity Study design DI-1859 study.**

| Group # | # of Mice (male/female) | Drug Route | Vol./ mouse | Test Article dose (mg/kg) | Treatment Schedule | Diluent |
|---|---|---|---|---|---|---|
| 1 | 5/0 | IP | 10µl/g | 0 mg/kg | qD(1-14) | 20% PCP |
| 2 | 5/0 | IP | 10µl/g | 50 mg/kg | qD(1-14) | 20% PCP |

**Supplementary Table 4A. Absolute organ weights (g) of mice treated (IP) with vehicle (Group 1) or DI-1859 at 50 mg/kg (Group 2). Data are presented as mean ± s.d. with n = 5.**

| Group | 1 | 2 |
|---|---|---|
| Dose (mg/kg) | 0 | 50 |
| Body weights | 26.56 ± 1.28 | 25.78 ± 1.11 |
| Brain | 0.465 ± 0.010 | 0.454 ± 0.009 |
| Heart | 0.120 ± 0.015 | 0.117 ± 0.009 |
| Liver[b] | 1.429 ± 0.102 | 1.596 ± 0.098 |
| L kidney | 0.184 ± 0.011 | 0.182 ± 0.013 |
| R kidney | 0.180 ± 0.015 | 0.200 ± 0.007 |
| Spleen | 0.070 ± 0.016 | 0.073 ± 0.013 |
| Thymus[b] | 0.035 ± 0.008 | 0.040 ± 0.027 |
| Adrenal glands (paired)[b,c] | 0.007 ± 0.002 | 0.006 ± 0.002 |
| Testes (paired) | 0.199 ± 0.014 | 0.222 ± 0.017 |
| Epididymis (paired) | 0.098 ± 0.015 | 0.089 ± 0.011 |

[a]Mean ±S.D. Comparison of Groups 2 to Group l (vehicle) by one-way ANOVA with Dunnet's correction except where noted. Significant differences from vehicle group indicated by asterisk.
[b]Analysis performed using Kruskal-Wallis with Dunn's correction due to non-normal distribution that could not be corrected by log transformation.
[c] One outlier value removed (false discovery=1%)
[d]Analysis performed on log-transformed values due to non-normal distribution.

**Supplementary Table 4B. Relative organ weights (% of body weight) of mice treated (IP) with vehicle (Group 1) or DI-1859 at 50 mg/kg (Group 2). Data are presented as mean ± s.d. with n = 5.**

| Group | 1 | 2 |
|---|---|---|
| Dose (mg/kg) | 0 | 50 |
| Brain | 1.76 ± 0.09 | 1.76 ± 0.07 |
| Heart | 0.45 ± 0.05 | 0.45 ± 0.02 |
| Liver[b] | 5.38 ± 0.19 | 6.19 ± 0.19* (p=0.0392) |
| L kidney | 0.69 ± 0.06 | 0.71 ± 0.05 |
| R kidney | 0.68 ± 0.04 | 0.78 ± 0.04* (p=0.0017) |
| Spleen | 0.26 ± 0.05 | 0.28 ± 0.04 |
| Thymus[b] | 0.13 ± 0.03 | 0.16 ± 0.11 |
| Adrenal glands (paired) | 0.03 ± 0.01 | 0.02 ± 0.01 |
| Testes (paired)[b] | 0.75 ± 0.04 | 0.86 ± 0.09 |
| Epididymis (paired) | 0.37 ± 0.04 | 0.35 ± 0.05 |

[a]Mean ±S.D. Comparison of Groups 2 to Group l (vehicle) by one-way ANOVA with Dunnet's correction except where noted. Significant differences from vehicle group indicated by asterisk.
[b]Analysis performed using Kruskal-Wallis with Dunn's correction due to non-normal distribution that could not be corrected by log transformation.
[c]One outlier value removed (false discovery=1%)

**Supplementary Table 5A. Hematology results[a] of mice treated (IP) with vehicle (Group 1) or DI-1859 at 50 mg/kg (Group 2). Data are presented as mean ± s.d. with n = 5.**

| Group # | 1 | 2 |
|---|---|---|
| Dose (mg/kg) | 0 | 50 |
| WBC (10e3/ul) | 7.848 ± 6.038 | 6.868 ± 3.993 |
| Neutrophils (10e3/ul) | 1.562 ± 1.206 | 1.650 ± 1.106 |
| Lymphocytes (10e3/ul) | 5.344 ± 4.274 | 4.568 ± 1.146 |
| Monocytes (10e3/ul) | 0.576 ± 0.468 | 0.422 ± 0.208 |
| Eosinophils (10e3/ul)[b] | 0.282 ± 0.310 | 0.178 ± 0.206 |
| Basophils (10e3/ul) | 0.082 ± 0.083 | 0.058 ± 0.052 |
| Neutrophils (%) | 20.33 ± 6.36 | 22.33 ± 4.41 |
| Lymphocytes (%) | 68.17 ± 9.70 | 68.03 ± 5.49 |
| Monocytes (%) | 7.66 ± 2.22 | 6.68 ± 1.64 |
| Eosinophils (%) | 2.97 ± 1.54 | 2.19 ± 1.94 |
| Basophils (%) | 0.89 ± 0.57 | 0.77 ± 0.61 |
| RBC (10e6/ul) | 8.44 ± 2.04 | 8.36 ± 0.67 |
| Hemoglobin (g/dL) | 12.04 ± 2.70 | 11.70 ± 1.06 |
| Hematocrit (%) | 38.02 ± 9.89 | 35.58 ± 3.02 |
| MCV (fL) | 44.88 ± 0.92 | 42.60 ± 0.66 *(p=0.0029) |
| MCH (pg)[c] | 14.36 ± 0.72 | 13.98 ± 0.39 |
| MCHC (g/dL) | 31.96 ± 1.93 | 32.88 ± 1.13 |
| RDW (%) | 17.38 ± 0.82 | 17.12 ± 0.43 |
| Platelets (10e3/uL) | 252.2 ± 132.5 | 585.6 ± 354.1 |
| MPV (fL) | 5.98 ± 0.59 | 5.06 ± 0.40 |

[a]Mean ±S.D. Statistical comparison of Groups 2 to Group l (vehicle) by one-way ANOVA with Dunnet's correction for multiple comparisons except where noted. Significant differences from vehicle group indicated by asterisk.

[b] Analysis performed on log-transformed values due to non-normal distribution.

[c] Analysis performed using Kruskal-Wallis with Dunn's correction due to non-normal distribution that could not be corrected by log transformation.

**Supplementary Table 5B. Serum chemistry results[a] of mice treated (IP) with vehicle (Group 1) or DI-1859 at 50 mg/kg (Group 2). Data are presented as mean ± s.d. with n = 5.**

| Group # | 1 | 2 |
|---|---|---|
| Dose (mg/kg) | 0 | 50 |
| AST (U/L) | 311 ± 179[b] | 50.8 ± 10.4 *(p=0.0043) |
| ALT (U/L)[c] | 131.7 ± 52.2[b] | 28.2 ± 4.1 *(p=0.0006) |

[a]Mean ±S.D. Statistical comparison of Groups 2 to Group l (vehicle) by one-way ANOVA with Dunnet's correction for multiple comparisons. Significant differences from vehicle group indicated by asterisk with p values given.

[b] 2 animals from group missing data due to insufficient volume for analysis

**Supplementary Table 6. Microscopic findings of mice treated (IP) with vehicle (Group 1) or DI-1859 at 50 mg/kg (Group 2).**

| | | (# affected/ # evaluated)[b,c] | |
|---|---|---|---|
| Tissue | Finding[a] | Group 1 (vehicle) | Group 2 (50mg/kg) |
| Testis | Spermatid retention | 0/10 | 10/10 (mild) |
| | Increased residual bodies | 0/10 | 10/10 (mild) |
| Epididymis | Increased exfoliated germ cells | 0/10 | 10/10 (mild) |
| | Sperm granuloma | 1/10 | 0/10 |
| Liver | Microgranulomas (mixed leukocyte infiltration, multifocal) | 2/5 (mild) | 1/5 (mild) |
| | Necrosis, random | 1/5 (mild) | 0/5 |
| Lung | none | 0/5 | 0/5 |
| Heart | none | 0/5 | 0/5 |
| Kidneys | none | 0/10 | 0/10 |
| Adrenal Glands | none | 0/9[c] | 0/8[c] |
| Urinary bladder | none | 0/5 | 0/5 |
| Stomach | none | 0/5 | 0/5 |
| Small intestine | none | 0/5 | 0/5 |
| Pancreas | none | 0/5 | 0/5 |
| Cecum | none | 0/5 | 0/5 |
| Colon | none | 0/4[c] | 0/5 |
| Spleen | none | 0/5 | 0/5 |
| Thymus | none | 0/5 | 0/5 |
| Mesenteric lymph node | none | 0/5 | 0/5 |
| Sternal bone marrow | none | 0/5 | 0/5 |

a. Compound-related findings highlighted.

b. Severity scores indicated in parentheses after incidence numbers.

c. Number of total evaluated tissues for the organ was decreased where indicated due to lack of sufficient tissue in the plane of section.

## SUPPLEMENTARY METHODS

### Chemistry Methods

**General Information**. The reactions were performed under an N2 atmosphere in anhydrous solvents, and the commercial reagents and solvents were used as supplied without further purification. The final products were purified by reverse phase HPLC (RP-HPLC) with water (0.1% of TFA) and CH3CN (0.1% of TFA) as eluents with a flow rate of 60 mL/min and the final compounds were >95% pure. Proton nuclear magnetic resonance (1H NMR) and carbon nuclear magnetic resonance (13C NMR) spectroscopy were performed on Bruker Advance 400 NMR spectrometers and chemical shifts are reported in parts per million (ppm) relative to an internal standard. NMR experiments were processed with Bruker Topspin (version 3.2) and analyzed with MestReNova software (version 11). High resolution mass spectra (HRMS) were obtained from Agilent Q-TOF Electrospray mass spectrometer.

**Synthetic procedures and characterization of compounds 1-12.**

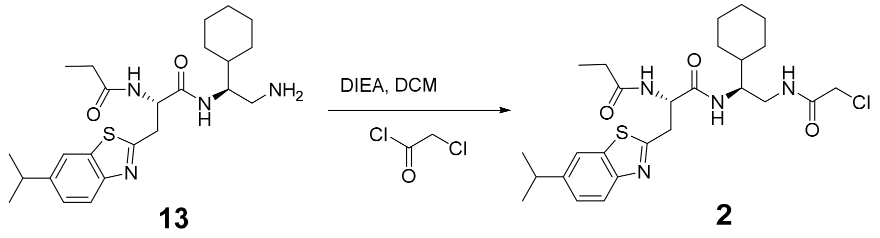


***(S)*-*N*-((*S*)-2-(2-chloroacetamido)-1-cyclohexylethyl)-3-(6-isopropylbenzo[*d*]thiazol-2-yl)-2-propionamidopropanamide (2):** Compound **13** was synthesized as previously described.[1] Chloroacetyl chloride (11 mg, 0.09 mmol, 1equiv.) was added to a solution of compound **13** (40 mg, 0.09 mmol, 1 equiv.) and DIEA (47 µL, 0.27 mmol, 3 equiv.) in DCM (5 mL) at 0 ºC. The resultant mixture was then stirred at room temperature for 1 h and concentrated. The residue was purified by HPLC to afford the compound **2** (33 mg, 71%). $^{1}$H NMR (400 MHz, $CDCl_3$) δ 7.85

(d, *J* = 8.4 Hz, 1H), 7.70 (s, 1H), 7.45 (d, *J* = 7.0 Hz, 1H), 7.37 (dd, *J* = 8.4, 1.2 Hz, 1H), 7.23-=7.14(m, 1H), 7.02 (d, *J* = 8.8 Hz, 1H), 4.95 (dd, *J* = 11.3, 6.3 Hz, 1H), 4.06 (d, *J* = 14.9 Hz, 1H), 3.94 (d, *J* = 15.0 Hz, 1H), 3.90 – 3.79 (m, 1H), 3.69 (dd, *J* = 15.6, 4.5 Hz, 1H), 3.48-3.28 (m, 3H), 3.06 (dt, *J* = 13.7, 6.8 Hz, 1H), 2.38 (q, *J* = 7.5 Hz, 2H), 1.69 – 1.47 (m, 5H), 1.39 – 1.28 (m, 7H), 1.22 (t, *J* = 7.6 Hz, 3H), 1.15 – 0.77 (m, 5H). $^{13}$C NMR (101 MHz, $CDCl_3$) δ 174.27, 170.85, 167.29, 166.83, 150.82, 146.74, 135.05, 125.62, 121.92, 118.83, 54.44, 52.24, 42.52, 41.72, 40.21, 35.32, 34.26, 29.66, 29.44, 28.39, 26.02, 25.95, 25.87, 24.17, 9.53. HRMS (ESI-MS) *m/z*: calculated for $C_{26}H_{38}ClN_4O_3S^+$ 521.2348, found 521.2350 $[M+H]^+$.

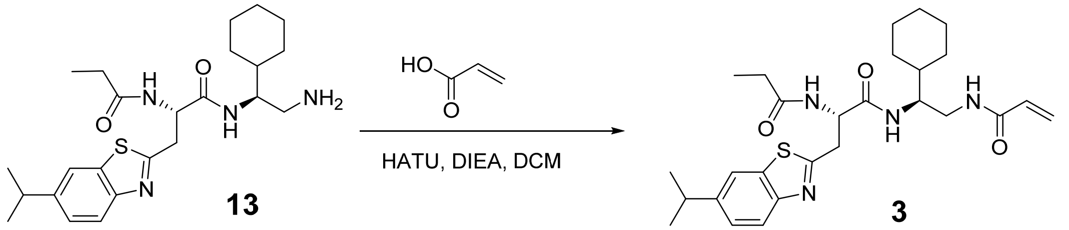


***N*-((*S*)-2-cyclohexyl-2-((*S*)-3-(6-isopropylbenzo[*d*]thiazol-2-yl)-2-propionamidopropanamido)ethyl)acrylamide (3 ):** Compound **13** (40 mg, 0.09 mmol, 1 equiv.) was added to a solution of acrylic acid (6.3 mg, 0.09 mmol, 1equiv.), HATU (51 mg, 0.13 mmol, 1.5 equiv.) and DIEA (47 µL, 0.27 mmol, 3 equiv.) in DCM (5 mL). The resultant mixture was stirred at room temperature for 1 h and concentrated. The residue was purified by HPLC to afford compound **3** (36 mg, 80%). $^{1}$H NMR (400 MHz, $CDCl_3$) δ 7.85 (d, *J* = 8.4 Hz, 1H), 7.71 (d, *J* = 1.2 Hz, 1H), 7.50 – 7.31 (m, 2H), 6.96-6.94 (m, 2H), 6.24 (dd, *J* = 17.0, 1.3 Hz, 1H), 6.09 (dd, *J* = 17.0, 10.2 Hz, 1H), 5.58 (dd, *J* = 10.2, 1.2 Hz, 1H), 4.94-4.90 (m, 1H), 3.94 – 3.76 (m, 1H), 3.64 (dd, *J* = 15.3, 4.8 Hz, 1H), 3.57 – 3.42 (m, 2H), 3.39 – 3.22 (m, 1H), 3.06 (dt, *J* = 13.8, 6.9 Hz, 1H), 2.36 (q, *J* = 7.6 Hz, 2H), 1.66-1.61 (m, 5H), 1.45 – 1.27 (m, 7H), 1.25 –

0.83 (m, 8H). $^{13}$C NMR (101 MHz, $CDCl_3$) δ 174.43, 170.68, 166.83, 166.52, 150.40, 146.95, 134.95, 130.71, 126.46, 125.82, 121.80, 118.87, 54.66, 52.63, 41.77, 40.08, 35.45, 34.27, 29.62, 29.52, 28.58, 26.07, 25.95, 25.89, 24.16, 9.61. HRMS (ESI-MS) *m/z*: calculated for $C_{27}H_{39}N_4O_3S^+$ 499.2737, found 499.2741 $[M+H]^+$.

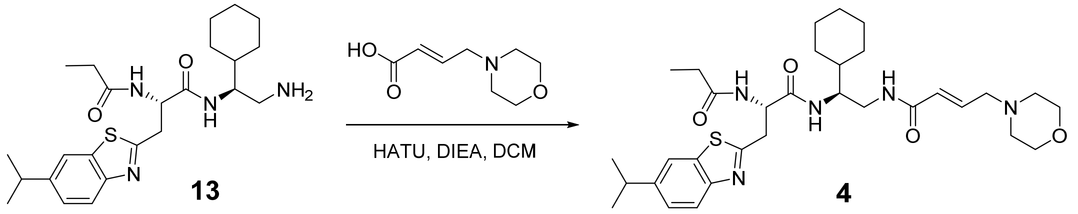


**(*E*)-*N*-((*S*)-2-cyclohexyl-2-((S)-3-(6-isopropylbenzo[*d*]thiazol-2-yl)-2-propionamidopropanamido)ethyl)-4-morpholinobut-2-enamide (4):** Compound **4** was prepared from **13** in 83% yield by a similar procedure as that for compound **3**. $^1$H NMR (400 MHz, MeOD) δ 7.86 (d, *J* = 8.5 Hz, 1H), 7.82 (d, *J* = 1.6 Hz, 1H), 7.42 (dd, *J* = 8.5, 1.7 Hz, 1H), 6.70 (dt, *J* = 15.0, 7.4 Hz, 1H), 6.37 (d, *J* = 15.3 Hz, 1H), 4.85 (dd, *J* = 8.2, 5.1 Hz, 1H), 4.16 – 3.91 (m, 4H), 3.85-3.76 (m, 3H), 3.59 (dd, *J* = 15.3, 5.1 Hz, 1H), 3.54-3.43 (m, 4H), 3.31 – 3.01 (m, 4H), 2.31 (q, *J* = 7.6 Hz, 2H), 1.73-1.61 (m, 5H), 1.49 – 1.39 (m, 1H), 1.32 (d, *J* = 6.9 Hz, 6H), 1.25 – 0.90 (m, 8H). $^{13}$C NMR (101 MHz, MeOD) δ 175.78, 171.38, 167.09, 165.21, 150.95, 146.70, 135.23, 132.88, 129.03, 125.23, 121.57, 118.61, 63.70, 56.84, 54.48, 53.08, 51.58, 40.53, 39.81, 35.10, 34.08, 29.52, 28.68, 28.34, 25.89, 25.73, 25.67, 23.17, 23.15, 8.73. HRMS (ESI-MS) *m/z*: calculated for $C_{32}H_{48}N_5O_4S^+$ 598.3422, found 598.3425 $[M+H]^+$.

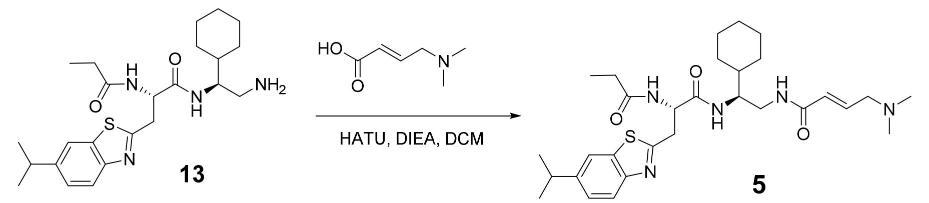

**(*E*)-*N*-((*S*)-2-cyclohexyl-2-((*S*)-3-(6-isopropylbenzo[d]thiazol-2-yl)-2-propionamidopropanamido)ethyl)-4-(dimethylamino)but-2-enamide (5):** Compound **5** was prepared from **13** in 78% yield by a similar procedure as that for compound **3**. $^1$H NMR (400 MHz, MeOD) δ 7.85 (d, *J* = 8.5 Hz, 1H), 7.82 (d, *J* = 1.7 Hz, 1H), 7.42 (dd, *J* = 8.5, 1.7 Hz, 1H), 6.76 – 6.60 (m, 1H), 6.35 (dt, *J* = 15.3, 1.1 Hz, 1H), 4.86 – 4.82 (m, 1H), 3.90 (dd, *J* = 7.3, 1.2 Hz, 2H), 3.85-3.80 (m, 1H), 3.59 (dd, *J* = 15.3, 5.1 Hz, 1H), 3.53 (dd, *J* = 13.7, 4.0 Hz, 1H), 3.46 (dd, *J* = 15.3, 8.2 Hz, 1H), 3.32 – 3.24 (m, 1H), 3.11-3.04 (m, 1H), 2.90 (s, 6H), 2.40 – 2.23 (m, 2H), 1.81 – 1.56 (m, 5H), 1.52 – 1.39 (m, 1H), 1.32 (d, *J* = 6.9 Hz, 6H), 1.25 – 0.88 (m, 8H). $^{13}$C NMR (101 MHz, MeOD) δ 175.79, 171.38, 167.10, 165.28, 150.94, 146.69, 135.22, 132.45, 129.76, 125.22, 121.56, 118.61, 57.41, 54.49, 53.07, 41.81, 40.56, 39.80, 35.06, 34.09, 29.52, 28.67, 28.33, 25.89, 25.73, 25.67, 23.15, 8.72. HRMS (ESI-MS) *m/z*: calculated for $C_{30}H_{46}N_5O_3S^+$ 556.3316, found 556.3316 [M+H]$^+$.

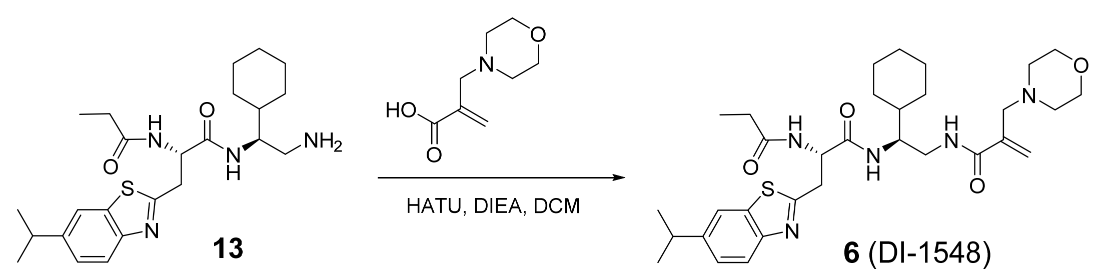

***N*-((*S*)-2-cyclohexyl-2-((*S*)-3-(6-isopropylbenzo[*d*]thiazol-2-yl)-2-propionamidopropanamido)ethyl)-2-(morpholinomethyl)acrylamide (6, DI-1548):**

Compound **6** (DI-1548) was prepared from **13** in 84% yield by a similar procedure as that for compound **3**. $^{1}$H NMR (400 MHz, MeOD) δ 7.85 (d, *J* = 8.5 Hz, 1H), 7.82 (d, *J* = 1.7 Hz, 1H), 7.42 (dd, *J* = 8.5, 1.7 Hz, 1H), 6.20 (s, 1H), 5.96 (s, 1H), 4.85 (dd, *J* = 7.7, 5.5 Hz, 1H), 4.05-3.95 (m, 4H), 3.90-3.85 (m, 3H), 3.62 (dd, *J* = 15.3, 5.5 Hz, 1H), 3.54-3.43 (ddd, *J* = 23.1, 14.5, 5.6 Hz, 4H), 3.27 – 3.02 (m, 4H), 2.34 (qd, *J* = 7.6, 0.9 Hz, 2H), 1.78 – 1.57 (m, 5H), 1.49 – 1.38 (m, 1H), 1.32 (d, *J* = 6.9 Hz, 6H), 1.25 – 0.86 (m, 8H). $^{13}$C NMR (101 MHz, MeOD) δ 175.94, 171.46, 167.39, 167.10, 150.97, 146.70, 135.18, 132.79, 129.05, 125.26, 121.60, 118.63, 63.49, 58.27, 54.37, 53.19, 51.77, 41.25, 39.75, 34.80, 34.08, 29.59, 28.76, 28.48, 25.89, 25.68, 25.60, 23.20, 8.79. HRMS (ESI-MS) *m/z*: calculated for $C_{32}H_{48}N_5O_4S^+$ 598.3422, found 598.3427 $[M+H]^+$.

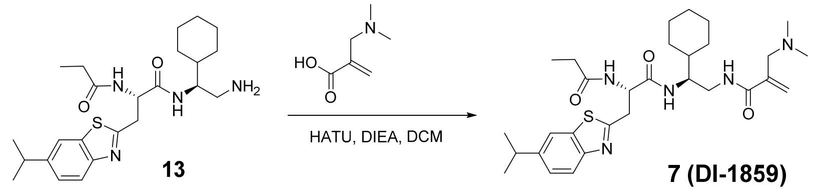


**N-((S)-2-cyclohexyl-2-((S)-3-(6-isopropylbenzo[d]thiazol-2-yl)-2-propionamidopropanamido)ethyl)-2-((dimethylamino)methyl)acrylamide (7, DI-859):**

Compound **7** (DI-1859) was prepared from **13** in 72% yield by a similar procedure as that for compound **3**. $^{1}$H NMR (400 MHz, MeOD) δ 7.85 (d, *J* = 8.5 Hz, 1H), 7.82 (d, *J* = 1.7 Hz, 1H), 7.41 (dd, *J* = 8.5, 1.7 Hz, 1H), 6.18 (s, 1H), 5.93 (s, 1H), 4.86 (dd, *J* = 7.7, 5.5 Hz, 1H), 3.99 – 3.82 (m, 3H), 3.62 (dd, *J* = 15.3, 5.5 Hz, 1H), 3.53-3.43 (m, 2H), 3.24 (dd, *J* = 13.7, 10.3 Hz, 1H), 3.07 (dt, *J* = 13.8, 6.9 Hz, 1H), 2.88 (s, 6H), 2.34 (qd, *J* = 7.6, 1.5 Hz, 2H), 1.79 – 1.57 (m, 5H), 1.47 – 1.38 (m, 1H), 1.32 (d, *J* = 6.9 Hz, 6H), 1.24 – 0.88 (m, 8H). $^{13}$C NMR (101 MHz, MeOD) δ 175.17, 170.62, 166.49, 166.30, 150.15, 145.89, 134.37, 132.78, 127.28, 124.44,

120.76, 117.80, 58.60, 53.62, 52.26, 41.33, 40.31, 38.94, 34.00, 33.28, 28.79, 27.89, 27.61, 25.07, 24.86, 24.78, 22.36, 7.89. HRMS (ESI-MS) *m/z*: calculated for $C_{30}H_{46}N_5O_3S^+$ 556.3316, found 556.3321 $[M+H]^+$.

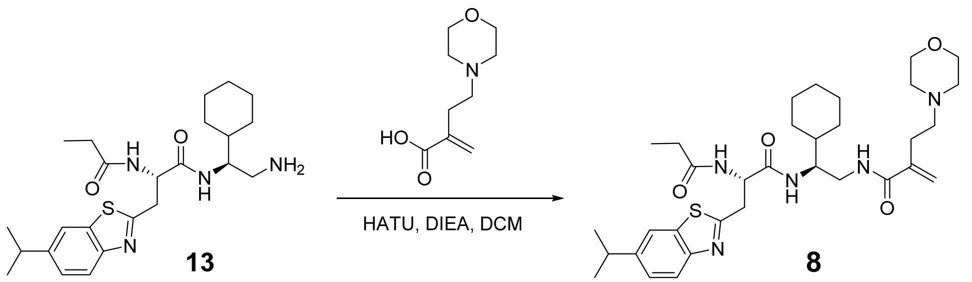


***N*-((*S*)-2-cyclohexyl-2-((*S*)-3-(6-isopropylbenzo[*d*]thiazol-2-yl)-2-propionamidopropanamido)ethyl)-2-methylene-4-morpholinobutanamide (8):** Compound **8** was prepared from **13** in 78% yield by a similar procedure as that for compound **3**. $^1H$ NMR (400 MHz, MeOD) δ 7.85 (d, $J$ = 8.5 Hz, 1H), 7.83 (d, $J$ = 1.7 Hz, 1H), 7.42 (dd, $J$ = 8.5, 1.7 Hz, 1H), 5.77 (s, 1H), 5.53 (s, 1H), 4.86 (dd, $J$ = 7.7, 5.9 Hz, 1H), 4.09-4.05 (m, 2H), 3.95-3.91 (m, 1H), 3.82-3.75 (m, 2H), 3.66 – 3.51 (m, 4H), 3.43 (dd, $J$ = 15.3, 7.8 Hz, 1H), 3.24 (t, $J$ = 7.0 Hz, 2H), 3.19 – 3.04 (m, 4H), 2.76-2.69 (m, 1H), 2.67 – 2.56 (m, 1H), 2.30 (q, $J$ = 7.6 Hz, 2H), 1.79 – 1.60 (m, 5H), 1.47-1.41 (dm, 1H), 1.33 (d, $J$ = 6.9 Hz, 6H), 1.26 – 0.92 (m, 8H). $^{13}C$ NMR (101 MHz, MeOD) δ 175.78, 171.46, 169.82, 167.16, 150.92, 146.71, 140.20, 135.23, 125.25, 122.03, 121.57, 118.64, 63.88, 57.03, 53.79, 53.06, 52.03, 51.90, 41.43, 39.70, 34.94, 34.08, 29.63, 28.64, 28.46, 27.08, 25.93, 25.67, 25.60, 23.19, 8.79. HRMS (ESI-MS) *m/z*: calculated for $C_{33}H_{50}N_5O_4S^+$ 612.3578, found 612.3583 $[M+H]^+$.

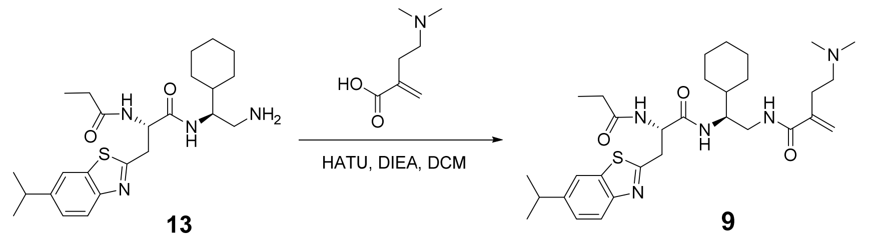

**$N$-(($S$)-2-cyclohexyl-2-(($S$)-3-(6-isopropylbenzo[$d$]thiazol-2-yl)-2-propionamidopropanamido)ethyl)-4-(dimethylamino)-2-methylenebutanamide (9):** Compound **9** was prepared from **13** in 80% yield by a similar procedure as that for compound **3**. $^1$H NMR (400 MHz, MeOD) δ 7.85 (d, $J$ = 8.5 Hz, 1H), 7.82 (d, $J$ = 1.7 Hz, 1H), 7.46 – 7.38 (m, 1H), 5.76 (s, 1H), 5.52 (s, 1H), 4.87 – 4.84 (m, 1H), 3.92-3.87 (m, 1H), 3.66 – 3.52 (m, 2H), 3.44 (dd, $J$ = 15.3, 7.8 Hz, 1H), 3.23 (t, $J$ = 7.2 Hz, 2H), 3.18-3.04 (m, 2H), 2.93 (s, 6H), 2.79 – 2.56 (m, 2H), 2.31 (q, $J$ = 7.6 Hz, 2H), 1.79 – 1.57 (m, 5H), 1.51 – 1.39 (m, 1H), 1.32 (d, $J$ = 6.9 Hz, 6H), 1.26 – 0.89 (m, 8H). $^{13}$C NMR (101 MHz, MeOD) δ 175.80, 171.46, 169.54, 167.11, 150.96, 146.71, 140.04, 135.22, 125.23, 121.69, 121.56, 118.62, 57.43, 54.03, 53.03, 42.26, 41.34, 39.76, 34.89, 34.09, 29.62, 28.65, 28.42, 27.98, 25.91, 25.70, 25.63, 23.16, 8.76. HRMS (ESI-MS) $m/z$: calculated for $C_{31}H_{48}N_5O_3S^+$ 570.3472, found 570.3474 $[M+H]^+$.

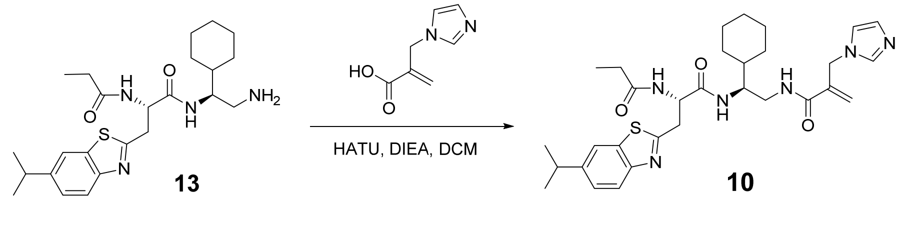

**2-((1H-imidazol-1-yl)methyl)-N-((S)-2-cyclohexyl-2-((S)-3-(6-isopropylbenzo[d]thiazol-2-yl)-2-propionamidopropanamido)ethyl)acrylamide (10):** Compound **10** was prepared from **13** in 77% yield by a similar procedure as that for compound **3**. $^1$H NMR (400 MHz, MeOD) δ 8.94

(t, *J* = 1.4 Hz, 1H), 7.91 – 7.75 (m, 2H), 7.62 – 7.56 (m, 1H), 7.56 – 7.50 (m, 1H), 7.46 – 7.37 (m, 1H), 6.04 (s, 1H), 5.78 (s, 1H), 5.06 (q, *J* = 14.7 Hz, 2H), 4.85-4.83 (m, 1H), 3.83-3.78 (m, 1H), 3.58 (dd, *J* = 15.2, 5.8 Hz, 1H), 3.50 (dd, *J* = 13.7, 3.7 Hz, 1H), 3.42 (dd, *J* = 15.2, 7.8 Hz, 1H), 3.20 – 3.02 (m, 2H), 2.30 (q, *J* = 7.6 Hz, 2H), 1.80 – 1.54 (m, 5H), 1.42 – 1.35 (m, 1H), 1.33 (d, *J* = 6.9 Hz, 6H), 1.26 – 0.84 (m, 8H). $^{13}$C NMR (101 MHz, MeOD) δ 175.77, 171.34, 167.08, 166.67, 150.97, 146.69, 137.93, 135.70, 135.23, 125.21, 124.17, 122.01, 121.55, 119.66, 118.61, 54.22, 52.98, 50.10, 41.11, 39.69, 34.91, 34.09, 29.56, 28.63, 28.31, 25.89, 25.71, 25.65, 23.17, 23.15, 8.73. HRMS (ESI-MS) *m/z*: calculated for $C_{31}H_{43}N_6O_3S^+$ 579.3112, found 579.3112 [M+H]$^+$.

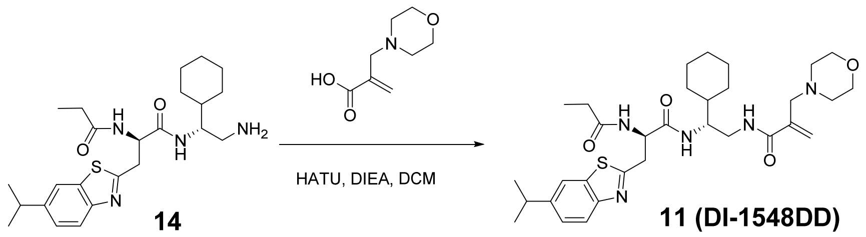


**N-((R)-2-cyclohexyl-2-((R)-3-(6-isopropylbenzo[d]thiazol-2-yl)-2-propionamidopropanamido)ethyl)-2-(morpholinomethyl)acrylamide (11, DI-548DD):** Compound **14** was synthesized as previously described.[1] Compound **14** (40 mg, 0.09 mmol, 1 equiv.) was added to a solution of 2-(4-morpholinylmethyl)acrylic acid (16 mg, 0.09 mmol, 1equiv.), HATU (51 mg, 0.13 mmol, 1.5 equiv.) and DIEA (47 µL, 0.27 mmol, 3 equiv.) in DCM (5 mL). The resultant mixture was stirred at room temperature for 1 h and concentrated. The residue was purified by HPLC to afford the compound **11** (43 mg, 79%). $^1$H NMR (400 MHz, MeOD) δ 7.86 (d, *J* = 8.5 Hz, 1H), 7.83 (d, *J* = 1.7 Hz, 1H), 7.43 (dd, *J* = 8.5, 1.7 Hz, 1H), 6.20 (s, 1H), 5.96 (s, 1H), 4.84 (dd, *J* = 7.7, 5.5 Hz, 1H), 4.05-3.95 (m, 4H), 3.90-3.85 (m, 3H),

3.61 (dd, $J$ = 15.4, 5.5 Hz, 1H), 3.54-3.43 (m, 4H), 3.26 – 3.03 (m, 4H), 2.35 (qd, $J$ = 7.6, 0.9 Hz, 2H), 1.77 – 1.59 (m, 5H), 1.425-1.39 (m, 1H), 1.33 (d, $J$ = 6.9 Hz, 6H), 1.25 – 0.87 (m, 8H). $^{13}$C NMR (101 MHz, MeOD) δ 175.98, 171.48, 167.40, 167.03, 151.02, 146.74, 135.17, 132.70, 128.90, 125.26, 121.59, 118.62, 63.48, 58.55, 54.42, 53.15, 51.82, 41.28, 39.76, 34.71, 34.09, 29.61, 28.75, 28.47, 25.87, 25.65, 25.58, 23.16, 23.15, 8.75. HRMS (ESI-MS) *m/z*: calculated for $C_{32}H_{48}N_5O_4S^+$ 598.3422, found 598.3424 [M+H]$^+$.

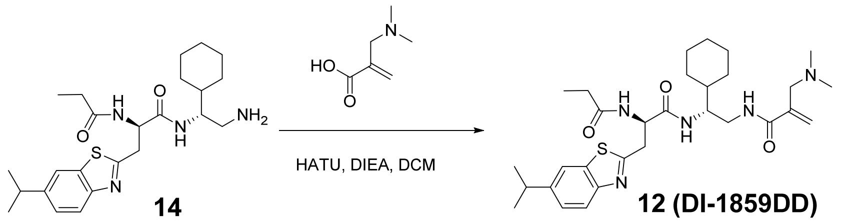


**N-((R)-2-cyclohexyl-2-((R)-3-(6-isopropylbenzo[d]thiazol-2-yl)-2-propionamidopropanamido)ethyl)-2-((dimethylamino)methyl)acrylamide (12, DI-1859DD).** Compound **12** was prepared from **14** in 75% yield by a similar procedure as that for compound **11**. $^1$H NMR (400 MHz, MeOD) δ 7.85 (d, $J$ = 8.5 Hz, 1H), 7.83 (d, $J$ = 1.7 Hz, 1H), 7.42 (dd, $J$ = 8.5, 1.7 Hz, 1H), 6.18 (s, 1H), 5.92 (s, 1H), 4.85-4.81 (m, 1H), 3.98 – 3.80 (m, 3H), 3.61 (dd, $J$ = 15.3, 5.5 Hz, 1H), 3.55 – 3.41 (m, 2H), 3.24 (dd, $J$ = 13.6, 10.4 Hz, 1H), 3.07 (dt, $J$ = 13.8, 6.9 Hz, 1H), 2.88 (s, 6H), 2.34 (qd, $J$ = 7.6, 1.4 Hz, 2H), 1.76 – 1.59 (m, 5H), 1.48 – 1.38 (m, 1H), 1.33 (d, $J$ = 6.9 Hz, 6H), 1.23 – 0.88 (m, 8H). $^{13}$C NMR (101 MHz, MeOD) δ 175.99, 171.44, 167.29, 167.06, 151.02, 146.71, 135.19, 133.58, 128.02, 125.24, 121.58, 118.61, 59.48, 54.44, 53.05, 42.14, 41.14, 39.75, 34.78, 34.09, 29.61, 28.69, 28.42, 25.87, 25.66, 25.58, 23.16, 8.69. HRMS (ESI-MS) *m/z*: calculated for $C_{30}H_{46}N_5O_3S^+$ 556.3316, found 556.3319 [M+H]$^+$.
