# Supplementary material for: Selective inhibition of cullin 3 neddylation through covalent targeting DCN1 protects mice from acetaminophen-induced liver toxicity
Source: Nat Commun. 2021 May 11;12:2621. doi: 10.1038/s41467-021-22924-4 (PMC8113459; doi:10.1038/s41467-021-22924-4)
Supplement: Supplementary file 3 — Reporting summary [file 41467_2021_22924_MOESM3_ESM.pdf]

nature research

Corresponding author(s): Shaomeng Wang

Last updated by author(s): Mar 20, 2021

Reporting Summary

Nature Research wishes to improve the reproducibility of the work that we publish. This form provides structure for consistency and transparency in reporting. For further information on Nature Research policies, see our Editorial Policies and the Editorial Policy Checklist.

Statistics

For all statistical analyses, confirm that the following items are present in the figure legend, table legend, main text, or Methods section.

n/a	Confirmed	
☐	☒	The exact sample size (*n*) for each experimental group/condition, given as a discrete number and unit of measurement
☐	☒	A statement on whether measurements were taken from distinct samples or whether the same sample was measured repeatedly
☐	☒	The statistical test(s) used AND whether they are one- or two-sided<br>*Only common tests should be described solely by name; describe more complex techniques in the Methods section.*
☒	☐	A description of all covariates tested
☒	☐	A description of any assumptions or corrections, such as tests of normality and adjustment for multiple comparisons
☐	☒	A full description of the statistical parameters including central tendency (e.g. means) or other basic estimates (e.g. regression coefficient) AND variation (e.g. standard deviation) or associated estimates of uncertainty (e.g. confidence intervals)
☐	☒	For null hypothesis testing, the test statistic (e.g. *F*, *t*, *r*) with confidence intervals, effect sizes, degrees of freedom and *P* value noted<br>*Give P values as exact values whenever suitable.*
☒	☐	For Bayesian analysis, information on the choice of priors and Markov chain Monte Carlo settings
☒	☐	For hierarchical and complex designs, identification of the appropriate level for tests and full reporting of outcomes
☒	☐	Estimates of effect sizes (e.g. Cohen's *d*, Pearson's *r*), indicating how they were calculated

Our web collection on statistics for biologists contains articles on many of the points above.

Software and code

Policy information about availability of computer code

Data collection	Bruker TopSpin (Version 3.2) for NMR data. Coot (Version 0.8.9.2), Buster (Version 2.10.3), Molprobity (Version 4.4) and Pymol (Version 2.4.1) for Crystallization. GraphPad prism 7.
Data analysis	MestReNova (Version 11) was used for NMR analysis. ImageJ software (Version 1.52v) was used for densitometry analysis. Statistical analysis Using ClinCalc.com » Statistics » Sample Size Calculator.

For manuscripts utilizing custom algorithms or software that are central to the research but not yet described in published literature, software must be made available to editors and reviewers. We strongly encourage code deposition in a community repository (e.g. GitHub). See the Nature Research guidelines for submitting code & software for further information.

Data

Policy information about availability of data

All manuscripts must include a data availability statement. This statement should provide the following information, where applicable:

- Accession codes, unique identifiers, or web links for publicly available datasets
- A list of figures that have associated raw data
- A description of any restrictions on data availability

The pdb coordination files of compounds DI-1548, DI-1859, 4, 8, 9 and 10 in a complex with DCN1 are deposited in the Protein Data Bank with accession codes 6XOL, 6XOO, 6XOQ, 6XOM, 6XON and 6XOP, respectively. The source data underlying Figs. 1–7 are provided as a Source Data file.

Field-specific reporting

Please select the one below that is the best fit for your research. If you are not sure, read the appropriate sections before making your selection.

- [x] Life sciences
- [ ] Behavioural & social sciences
- [ ] Ecological, evolutionary & environmental sciences

For a reference copy of the document with all sections, see nature.com/documents/nr-reporting-summary-flat.pdf

Life sciences study design

All studies must disclose on these points even when the disclosure is negative.

Sample size	No sample size calculation was performed for this study. Sample size for animal study is 8-9 mice per group.
Data exclusions	Data are not excluded from analysis.
Replication	The biochemical and biophysical experiments performed with two or more replication. All attempts at replication were successful.
Randomization	Animals were randomly allocated into experimental groups. Cells were seeded in plates and randomly treated with various compounds with different concentrations and time points for analysis.
Blinding	Data collection of most mouse experiments (Blood analysis, H&E staining and ROS assays) were performed in a blinding manner. The biochemical and cellular experiments were not performed in a blinding manner.

Reporting for specific materials, systems and methods

We require information from authors about some types of materials, experimental systems and methods used in many studies. Here, indicate whether each material, system or method listed is relevant to your study. If you are not sure if a list item applies to your research, read the appropriate section before selecting a response.

Materials & experimental systems

n/a	Involved in the study	
[ ]	[x]	Antibodies
[ ]	[x]	Eukaryotic cell lines
[x]	[ ]	Palaeontology and archaeology
[ ]	[x]	Animals and other organisms
[x]	[ ]	Human research participants
[x]	[ ]	Clinical data
[x]	[ ]	Dual use research of concern

Methods

n/a	Involved in the study	
[x]	[ ]	ChIP-seq
[x]	[ ]	Flow cytometry
[x]	[ ]	MRI-based neuroimaging

Antibodies

Antibodies used	Cullin 1 (sc-17775, 1:500 dilution), and Cullin 2 (sc- 166506, 1:500 dilution), from Santa Cruz Biotech (Santa Cruz, CA); Cullin 4A (PA5-14542, 1:1000 dilution), Cullin 4B (PA5-50647, 1:1000 dilution) and DCN3 (DCUN1D3, PA5-44000, 1:1000 dilution) from ThermoFisher Scientific (Wayne, MI); Cullin 3 (2759, 1:1000), NRF2 (12721, 1:1000 dilution), HO-1 (70081, 1:1000 dilution) and Bim (2819, 1:1000 dilution) from Cell Signaling Technology (Boston, MA); DCN1 (GWB-E3D700, 1:1000 dilution) from GenWay Biotech (San Diego, CA). Cullin 5 (A302-173A, 1:500 dilution) from Bethyl Labs, Montgomery, TX).
Validation	Antibodies was validated by manufacturer: Cullin 1 (sc-17775, confirmed in HOS and JAR cell lines), and Cullin 2 (sc- 166506, confirmed in Raji and K-562 cell lines), from Santa Cruz Biotech (Santa Cruz, CA); Cullin 4A (PA5-14542, confirmed in 293 cells), Cullin 4B (PA5-50647, confirmed in HepG2 cell line) and DCN3 (DCUN1D3, PA5-44000, confirmed in human kidney cells) from ThermoFisher Scientific (Wayne, MI); Cullin 3 (2759, confirmed in COS and PC12 cell lines), NRF2 (12721, confirmed in MEF and U2OS cell lines), HO-1 (70081, confirmed in Hela and 3T3 cell lines) and Bim (2819, confirmed in Raji, A20 cell lines) from Cell Signaling Technology (Boston, MA); DCN1 (GWB-E3D700, confirmed by siRNA in our study) from GenWay Biotech (San Diego, CA). Cullin 5 (A302-173A, confirmed in HEK293T cell line) from Bethyl Labs, Montgomery, TX).

Eukaryotic cell lines

Policy information about cell lines

Cell line source(s)	U2OS, MDA-MA-231, HCT116, THLE2 cell lines were purchased from he American Type Culture Collection (ATCC) (Manassas, VA). KEYS70 was purchased from DSMZ (Braunschweig, Germany).

Authentication	Cell lines were directly purchased from vendors and the vendors performed the authentication. No further authentication was performed.
Mycoplasma contamination	None of the cells used was tested for mycoplasma contamination.
Commonly misidentified lines (See ICLAC register)	No misidentified cell line was used.

Animals and other organisms

Policy information about studies involving animals; ARRIVE guidelines recommended for reporting animal research

Laboratory animals	C57BL/6 WT mice (~8 weeks, male, normal chow) were purchased from The Jackson Laboratory. The mice were maintained under 68-79 °F ambient temperature, 30%-70% humidity and a 12 hour dark/light cycle.
Wild animals	This study did not involved wild animals.
Field-collected samples	This study did not involved samples collected from the filed.
Ethics oversight	The in vivo studies were performed under animal protocols (PRO00007499) and (PRO00006638) approved by the Institutional Animal Care & Use Committee (IACUC) of the University of Michigan, in accordance with the recommendations in the Guide for the Care and Use of Laboratory Animals of the National Institutes of Health.

Note that full information on the approval of the study protocol must also be provided in the manuscript.
